# The hierarchically mechanistic mind: an evolutionary systems theory of the human brain, cognition, and behavior

**DOI:** 10.3758/s13415-019-00721-3

**Published:** 2019-05-21

**Authors:** Paul B. Badcock, Karl J. Friston, Maxwell J. D. Ramstead, Annemie Ploeger, Jakob Hohwy

**Affiliations:** 1 Centre for Youth Mental Health, The University of Melbourne, Melbourne, Australia; 2 Melbourne School of Psychological Sciences, The University of Melbourne, Melbourne, Australia; 3 Orygen, The National Centre of Excellence in Youth Mental Health, Melbourne, Australia; 4 Wellcome Trust Centre for Neuroimaging, University College London, London, UK; 5 Department of Philosophy, McGill University, Montreal, QC Canada; 6 Division of Social and Transcultural Psychiatry, Department of Psychiatry, McGill University, Montreal, QC Canada; 7 Department of Psychology, University of Amsterdam, Amsterdam, The Netherlands; 8 Cognition & Philosophy Lab, Monash University, Clayton, VIC Australia

**Keywords:** Active inference, Adaptive prior, Developmental psychology, Evolutionary psychology, Evolutionary Systems Theory, Free-Energy Principle, Hierarchically Mechanistic Mind

## Abstract

The purpose of this review was to integrate leading paradigms in psychology and neuroscience with a theory of the embodied, situated human brain, called the Hierarchically Mechanistic Mind (HMM). The HMM describes the brain as a complex adaptive system that functions to minimize the entropy of our sensory and physical states via action-perception cycles generated by hierarchical neural dynamics. First, we review the extant literature on the hierarchical structure of the brain. Next, we derive the HMM from a broader evolutionary systems theory that explains neural structure and function in terms of dynamic interactions across four nested levels of biological causation (i.e., adaptation, phylogeny, ontogeny, and mechanism). We then describe how the HMM aligns with a global brain theory in neuroscience called the free-energy principle, leveraging this theory to mathematically formulate neural dynamics across hierarchical spatiotemporal scales. We conclude by exploring the implications of the HMM for psychological inquiry.

Since the turn of the century, we have made remarkable progress in our understanding of the human brain. This has been facilitated in particular by improvements in neuroimaging, coupled with analytical tools gleaned from mathematical modeling. Concurrently, there has been a growing appreciation of the fact that in order to elucidate the fundamental relationships between neural dynamics, structure, and function—and the brain, cognition, and behavior—cognitive scientists need to bridge intra- and interdisciplinary divisions by exploring promising points of contact between different paradigms. In psychology in particular, a fragmentation into disparate fields of inquiry has long been recognized as an impediment to progress (Buss, 1995; Henriques, 2011).

With this in mind, the purpose of this review is to suggest that many extant models of the structure, dynamics, and function of the brain can be integrated under the unifying framework of the Hierarchically Mechanistic Mind (HMM). Originally proposed to synthesize evolutionary and developmental psychology (Badcock, 2012), the HMM has since been leveraged to explain depression (Badcock, Davey, Whittle, N.B. Allen, & Friston, 2017) and forwarded as a biologically plausible model of the human brain and biobehavior for the life sciences (Badcock, Friston, & Ramstead, 2019; Ramstead, Badcock & Friston, 2018a, 2018b). Drawing chiefly from psychology and neuroscience, this model describes the brain as an embodied, *complex adaptive system* that actively minimizes the entropy (i.e., the spread or decay) of human sensory and physiological states by generating adaptive action-perception cycles via dynamic interactions between hierarchically organized, differentially integrated neural subsystems (Badcock et al., 2019).

Our paper comprises four sections. After providing an empirically informed description of the structural (hierarchical) *organization* of the brain in the first section, we introduce an evolutionary systems theory that explains the *origins* of this hierarchical organization in terms of causal interactions between the broader evolutionary, developmental, and biopsychosocial processes that shape human phenotypes. In the third, we submit that the ensuing perspective of the embodied brain aligns with the free-energy principle (FEP) in neuroscience. We leverage the FEP to supply a *formal theory* of the brain, which can be used to derive empirically tractable *process theories* of human neural dynamics. Briefly, the FEP describes the brain as an “inference machine” that optimizes the evidence for the statistical model of the world that it encodes or embodies, by minimizing an upper limit or bound on surprise (i.e., variational free-energy). These three sections of our treatment address complementary questions about the nature of the human brain: what is the organization of this system, how does it come to be, and why is it the way that it is? After bringing these themes together to precisely define the HMM, we conclude by exploring its implications for theorizing and research across the psychological sciences.

In summary, the HMM encapsulates: (1) an evolutionary systems model of the human brain based on complementary levels of analysis in the psychological sciences; and (2) a mathematical model for formulating dynamics at (and across) each of these levels, based on the FEP. Ultimately, we argue that an interdisciplinary framework that calls upon both of these approaches provides a more cohesive and powerful explanation for the brain and behavior than either of them alone. In and of itself, the FEP is simply an information-theoretic formulation of the adaptive, self-organizing dynamics of sentient systems; arguably, combining the FEP with theories and research spanning psychology will allow us to unpack systematically the various ways in which *Homo sapiens* exemplify this principle (Badcock et al., 2019; Ramstead et al., 2018a, 2018b). With these distinctions in mind, the HMM can be described as a *process theory* in two complementary ways: it applies the FEP to the multiscale dynamics of the embodied human brain and behavior; and it appeals to the nested evolutionary, developmental, and real-time processes captured by different levels of explanation in psychology. By combining these approaches, psychologists will be better equipped to unpack the complex relationships between the brain, our minds, and our behavior.

## The Hierarchical Architecture of the Human Brain

The HMM rests on the architectural claim that the brain is a hierarchically organized system of neurocognitive mechanisms that interact in a dynamic, bidirectional fashion and that vary in degrees of functional specialization and integration (Badcock et al., 2019). According to this scheme, the lowest levels of the cortical hierarchy comprise relatively segregated, specialized neural mechanisms responsible for sensorimotor processing (so-called “domain-specific” systems); while its higher levels comprise developmentally plastic, highly integrated (“domain-general”) mechanisms that respond flexibly to input provided by lower levels, feed information back for further processing, and underlie our executive cognitive functions (e.g., meta-cognition) (Badcock, 2012). Two key terms require clarification.

The first is “hierarchy.” There are many interpretations of the neural hierarchy, but the one that we refer to here is a *fractal* or *self-similar* hierarchy, which entails the repeated encapsulation of smaller (neural) elements in larger ones (Kaiser, Hilgetag & Kötter, 2010). This sort of hierarchical organization is recapitulated across multiple (spatial, temporal, topological, and functional) neural scales (Breakspear & Stam, 2005; Power et al., 2011). Otherwise, the HHM does not commit to any particular form of hierarchy (e.g., subsumption hierarchies). Although there is ample evidence from neurobiology for deep serial hierarchies in the cortex, there also are violations of a simple serial architecture. Obvious examples are cortical hierarchies (e.g., cortico-cortical projections) that are “crosscut” with cortico-subcortical hierarchies (e.g., corticothalamic and thalamocortical projections). Furthermore, even within serial cortical hierarchies there are anomalies. For example, the frontal eye fields are paradoxically low in the visual hierarchy—based on their forward and backward connectivity (Mejias et al., 2016). More generally, the key aspect of a hierarchy is the emergence and maintenance of the right sort of conditional dependencies (and implicit connectivity) that allow the joint expression of functional segregation and integration—and an implicit separation of temporal scales (Bullmore & Sporns, 2009; Friston & Buzsaki, 2016; Markov et al., 2013; Sporns et al., 2005).

The second term is “neurocognitive mechanism.” Here, we refer to a neural subsystem at any spatial scale—from a neuronal population through to macroscopic brain regions—that can be characterized by: (1) specialized functional processing mediated by dense, short-range connections intrinsic to that scale (i.e., its local integration); and (2) its global (functional) integration via relatively sparse, long-range (e.g., extrinsic cortico-cortical) connections (Park & Friston, 2013). Under this model, cognition emerges from the global integration of local, functionally differentiated neural processing mechanisms (Park & Friston, 2013). This definition implies a complementary relationship between functional segregation and integration: all neural subsystems comprise a subpopulation of cells that have a common, specialized function, but they also are integrated because of their connectivity with other subsystems (Friston, 2003; Park & Friston, 2005). In network neuroscience, this kind of subsystem is called a module (Sporns & Betzel, 2016).

It is important to clarify the difference between modularity as it is used in the network neuroscience community, where it refers to highly interconnected neural elements that are relatively sparsely connected to other modules in the network, and traditional notions of modularity stemming from evolutionary psychology, where it refers to separately modifiable, functional specializations sculpted by evolution (Barrett & Kurzban, 2006; Buss, 1995; Fodor, 1983). The HMM borrows directly from the former sense of “module,” not the latter. We suggest that distinct patterns of adaptive behavior depend as much on the functional integration of such modules as they do on the operation of any given one—a claim that does not sit well with massive modularity. The type of mechanism we refer to follows contemporary, neomechanistic approaches in the philosophy of science that seek to explain the properties, functions, and behavior of a system by elucidating the properties and organized activities of its subcomponents and their interactions (Craver, 2001, 2006; Piccinini & Craver, 2011). In this context, a mechanism is broadly defined as a structure within a system that performs a function through its component parts, the operations of these parts, and their organization, which contributes to global functioning in one or more ways (Bechtel, 2008). With respect to the brain, the term “mechanism” is simply synonymous with any neural “subsystem” or “process” that contributes to the dynamics of the system itself—be it the form and function of any given one (e.g., the amygdala), or the coordinated operations of interactions between them (e.g., the activity of the limbic system). A key property of such subsystems is hierarchical near-decomposability: they are hierarchically organized, and unlike informationally encapsulated modules, their functioning cannot be completely individuated from other subsystems (Bechtel, 2008; Simon, 1996). As will be seen, this is a hallmark feature of complex adaptive systems that flows directly from the complementary relationship between natural selection and self-organization.

In sum, the hierarchical architecture that we describe follows a widespread consensus in cognitive neuroscience that cognition emerges from the hierarchical dynamics of segregated neural processing mechanisms that operate in a functionally integrated, bidirectional fashion (Markov & Kennedy, 2013; Mesulam, 2012; Meunier, Lambiotte & Bullmore, 2010; C.J. Price & Friston, 2002). We turn now to the extensive theoretical and empirical support for this view.

## The Hierarchical Structure of the Brain: A Brief Review of the Empirical Evidence

In psychology, the hierarchical architecture of the brain has long been emphasized by two prevailing schools of thought. On the one hand, evolutionary psychologists—particularly proponents of massive modularity—have argued that the brain comprises a large collection of functionally specialized modules dedicated to solving specific adaptive problems (Barrett & Kurzban, 2006; Buss, 1995). Drawing on evidence from evolutionary developmental biology, genetics, brain mapping, and comparative studies, H.C. Barrett (2012) has argued that the sharp distinction between highly specialized, domain-specific modules and general-purpose, domain-general systems is a false dichotomy. Rather, functionally specialized modules are likely to be both heterogeneous and hierarchically organized. Likewise, others have proposed that the adapted mind entails a hierarchy of modules, ranging from lower-order psychobiological mechanisms characterized by automatic, serial processing, and a high degree of specialization, through to higher-level modules that are flexible in their responses to input and production of outputs, allow us to gain awareness of these outputs, and enable top-down cognitive control (Cundall, 2006; Geary, 2005; Geary & Huffman, 2002; Merritt, 2008).

On the other hand, developmental psychologists have traditionally espoused a constructivist view that explains the hierarchical organization of the brain in terms of the progressive, ontogenetic modularization of the cortex (Karmiloff-Smith, 1992). According to this process-focused scheme, human cortical development reflects the hierarchical construction of “mental representations,” which involves the progressive, experience-dependent elaboration of neural circuits from primary sensorimotor areas to higher, more combinatorially complex (association) regions (Quartz, 1999). This provides flexibility when faced with a dynamic environment, explains cortical plasticity throughout the lifespan, and produces the higher-order association cortices responsible for our executive cognitive faculties. Unlike massive modularity, this view maintains that infants begin with a limited set of innately specified, domain-specific predispositions, allowing recursive interactions between these low-level systems and the environment to produce the functional organization of the brain throughout development (Karmiloff-Smith, 1998).

Despite longstanding debates between these schools about the causal primacy of evolutionary versus developmental processes (Badcock, 2012; Caporael, 2001; Frankenhuis, Panchanathan, & Barrett, 2013), the hierarchical structure of the brain has remained a central claim of both. This idea is backed by a wealth of empirical support. By way of illustration, functional imaging work has shown that when attempting to ascribe mental states based on incongruent social cues, participants’ exposure to conflicting nonverbal versus verbal cues both engaged the anterior cingulate and lateral prefrontal cortex (components of a “domain-general” cognitive control system that resolves perceptual conflict by regulating “downstream” neural structures), while differentially recruiting two lower-order systems sensitive to different types of social stimuli: the mirror neuron system and mental state attribution system, respectively (Zaki, Hennington, Weber & Ochsner, 2010). Such results imply that hierarchical interactions between relatively segregated and integrated mechanisms are involved in specific cognitive domains (i.e., social cognition; also see Colombo, 2014; Merritt, 2008). Similar evidence has emerged from research on Theory of Mind (Gerrans & Stone, 2008), face recognition (Nakamura et al., 2000), speech (Doupe & Kuhl, 1999), and working memory (Hasson, Chen & Honey, 2015).

The idea that neurodevelopment produces a flexible network of nested, increasingly domain-general systems is further supported by large meta-analyses of neuroimaging data, which have shown that individual brain regions are functionally diverse and have different functional partners in different contexts (Anderson, 2014; Anderson, Kinnison, & Pessoa, 2013). Domain-general systems also have been identified by imaging studies showing that specific frontal and parietal regions are engaged by a wide variety of cognitively demanding tasks (Fedorenko, Duncan, & Kanwisher, 2013). On the other end of the spectrum, it has been found that even at the level of the sensorium, highly segregated “domain-specific” systems process information in an integrated, bidirectional fashion. This is exemplified by cross- and multi-modal context effects in early sensory processing, where responses to unimodal sensory input are affected by information processed by other sensory modalities, with latencies suggesting that inputs in one modality directly influence early responses to stimuli presented to another (Giard & Peronnet, 1999; Spence, 2011).

Taken together, the work above speaks to a growing consensus that neurocognitive mechanisms are organized and interact in a hierarchical, bidirectional manner. There is now extensive comparative evidence to suggest that this sort of architecture is a hallmark of the mammalian brain, progressing from highly segregated (subcortical, cerebellar, and sensorimotor) systems common to all mammals through to the highly interconnected cortical association areas (e.g., the default mode, salience, and control networks) found in primates (Buckner & Krienen 2013; Finlay & Uchiyama 2015; Gu et al., 2015; Markov & Kennedy 2013; Mesulam, 2012). These widely distributed systems integrate information across large areas of cortical input, subserve “internal mentation” and our remarkable cognitive abilities, and confer the adaptive advantage of heightened cognitive control (Buckner & Krienen 2013; Finlay & Uchiyama, 2015).

To date, however, the strongest evidence for a hierarchical neural architecture has stemmed from network neuroscience, which focuses on the distributed networks of neural populations and brain regions responsible for cognition and behavior (Sporns & Betzel, 2016). Following graph theory, a neural network is represented as a collection of nodes (i.e., individual neural elements or interacting units of the network) and edges (i.e., the connections between nodes), forming “modules” comprised of densely connected nodes (i.e., network communities) that are sparsely connected to other nodes in the network (Sporns & Betzel, 2016). Wide-ranging studies of structural and functional connectivity in the brain suggest that it is organized as a self-similar hierarchy: a given node (e.g., network, module or sub-module) comprises a network of smaller interacting nodes at a lower (hierarchical) level, ranging from macroscopic neural networks and brain regions through to macrocolumns and neurons (Breakspear & Stam, 2015; Kaiser et al., 2010; Meunier et al., 2010; Park & Friston, 2013; Sporns, 2013). Fine-grained functional connectivity studies have confirmed that a self-similar hierarchy allows cortical networks to optimize the balance between local, specialized processing and global integration, while high-resolution structural connectivity findings have furnished complementary evidence that specialized motor tasks have a structural (segregated or modular) counterpart (Hütt, Kaiser, & Hilgetag, 2014; Kaiser, 2017; Taylor, Wang, & Kaiser, 2017).

## The Functional Hierarchy of the Brain: Predictive Coding as a Theory of Neural Processing

How does this self-similar hierarchy relate to function? An answer to this question has arisen from *predictive coding* in neuroscience (Lee & Mumford, 2003; Rao & Ballard, 1999). This is an influential paradigm that sees the brain as a hierarchical inference machine, which minimizes prediction error by reducing discrepancies between incoming sensory inputs and top-down predictions (A. Clark, 2013). According to this perspective, the brain embodies a hierarchical generative model: its physical (internal) states encode a hierarchy of hypotheses about the world that reflects a probabilistic mapping from causes in the environment to observed consequences (e.g., sensory data). Conditional expectations are thought to be encoded by deep pyramidal cells (i.e., representation units) at each level of the cortical hierarchy that convey predictions downward to suppress errors at the level below, whereas prediction errors (or deviations from expectations) are encoded by superficial pyramidal cells (i.e., error units) that convey errors forward to revise expectations at the level above, thereby minimizing prediction error (Bastos et al., 2012; Brown, Adams, Parees, Edwards & Friston, 2013; Mumford, 1992). Prediction errors also are weighted by precisions, which determine the relative influence of ascending (error) and descending (representation) signals (e.g., a high precision on error signals corresponds to low confidence in top-down expectations). Dynamic precision weighting is thought to be mediated by neuromodulation and underwrites cognitive processes such as attentional selection and sensory attenuation.

Arguably, predictive coding affords a plausible process theory of the functional integration of hierarchically modular networks. According to this scheme, minimizing prediction error entails the dynamic, online adjustment of edge strengths (i.e., connectivity) within the network by changing synaptic efficiency, with backwards connections delivering predictions to lower levels, and forward connections conveying prediction errors to higher ones (Park & Friston, 2013). Intrinsic states and edge strengths are recursively revised to improve predictions at each level of the hierarchy, while directed edge strengths reflect the effective connectivity of a network (i.e., the directed causal relationships between modules or nodes) when engaged during a specific task (Park & Friston, 2013). Cognition can therefore be described as the global integration of local (i.e., segregated) neuronal operations via hierarchical (error minimizing) message passing between cortical areas, a process that is facilitated by a hierarchically modular network structure (Park & Friston, 2013).

## The HMM: An Evolutionary Systems Theory of the Embodied, Situated Human Brain

We have considered empirical evidence that the architecture of the brain comprises a modular hierarchy of differentially integrated neural subsystems. However, we have yet to relate this neural architecture with a broader perspective on the embodied human brain. How does this hierarchical organization emerge from the evolutionary and developmental dynamics of the human brain-body-environment system? What are the various causal mechanisms particular to *Homo sapiens* responsible for producing and influencing it? To address these questions, we will introduce a meta-theoretical approach to psychological inquiry based on evolutionary systems theory.

## Evolutionary Systems Theory: The Origins of the Brain

Evolutionary systems theory (EST) is a prominent, transdisciplinary paradigm that hearkens back to the musings of Schrödinger (1944) and rests upon the elegant principle of co-action between *general selection* and *self-organization* to explain the evolution, form, and functioning of any dynamic, multicomponent system over time (Badcock, 2012; Ramstead et al., 2018a).

Originating from biology, general selection is a nonsubstantive, Darwinian process that involves three interacting principles of change: variation, selection, and retention (Caporael, 2001). This is a universal process that extends across statistical and quantum mechanics (Ao, 2008, 2014; Campbell, 2016), which not only applies to organisms (i.e., natural, kin, and sexual selection) but acts on all dynamically coupled systems, such as molecules, neural synapses, ideas, cultural practices, and technological products (Caporael, 2001; Cziko, 1995; Mesoudi, Whiten & Laland, 2006). Conversely, self-organization stems from dynamic systems theory in physics (Nicolis & Prigogine, 1977; Prigogine & Stengers, 1984) and refers to the spontaneous emergence of coherent, higher-order patterns resulting from recursive interactions among the simpler components of a complex, dynamic system (Lewis, 2000). There are four key properties of self-organizing systems: (1) microscopic coordinations emerge between different components of the system that lead to new macroscopic patterns, which perform unique functions that entrain and reinforce particular lower-order patterns over time (a process of *circular causality* between different levels of the system; see Witherington, 2007); (2) on average, they become progressively complex and ordered over time; (3) global reorganizations toward complexity occur at *phase transitions*—points of turbulent instability that allow old patterns to be replaced by new ones; and (4) they are both stable and sensitive to environmental conditions: emergent change is stabilized through negative feedback loops and macroscopic functional coordinations, while an interconnectedness with other systems favors sensitivity to the environment, particularly during phase transitions (Lewis, 2000). Notably, the interrelationships between time and different levels of systemic organization mean that dynamic activity within any one timescale (e.g., neural activity) is continuous with, and nested within, the dynamics of all other timescales (e.g., learning, development, and evolution) (Ramstead et al., 2018a; Smith & Thelen, 2003). Thus, an important extension of this approach is the need to analyze dynamic interactions across timescales.

With these distinctions in mind, the central premise of EST is as follows: given that certain functional (global or macroscopic) patterns of interacting (local or microscopic) components are selected over competing alternatives to allow different hierarchical levels of (physical, chemical, biological, psychological, and sociocultural) organization to emerge, self-organization and general selection represent the two fundamental, mutually reinforcing processes that drive any evolving system (Badcock, 2012; Eigen & Schuster, 1979; Kauffman, 1993; Weber & Depew, 1996). Work in this area has mainly centered on *complex adaptive systems*—a type of dynamically coupled, self-organizing system that adapts to its environment. This adaptation involves an autonomous process of selection that recruits the outcomes of a diversity of locally interacting components within that system to select a subset of those components for replication or enhancement (Levin, 2003). Prominent examples include the immune system (Holland, 1995), social systems (Lansing, 2003; Miller & Page, 2009), ecosystems and the biosphere (Levin, 1998), and of particular interest here, the brain (Haken, 1996; Kelso, 1996).

The relative validity of Darwinian versus dynamical approaches has long fueled debate in psychology, with evolutionary psychologists favoring the former school and developmentalists the latter (Badcock, 2012; Barrett & Kurzban, 2006; Frankenhuis et al., 2013; Greenberg, Partridge, Mosack, & Lambdin, 2006; Lickliter & Honeycutt, 2003). More recently, however, there has been growing advocacy of a dialectical approach that synthesizes these perspectives (Badcock, 2012; Frankenhuis et al., 2013; Kenrick, 2001; Ploeger et al., 2008a). Similarly, the model of the brain that we present is premised on the notion that these approaches are commensurate and complementary. Whereas evolutionary hypotheses address the ultimate questions of psychology by focusing on the adaptive properties of cognition and behavior, developmental systems approaches address its proximate questions by illuminating the ontogenetic and real-time processes responsible for producing them (Badcock, Ploeger, & Allen, 2016; Kenrick, 2001; Ramstead et al., 2018a). This line of thought resonates with growing evidence in biology that ultimate and proximate causes have a recursive, bidirectional relationship (Laland, Sterelny, Odling-Smee, Hoppitt & Uller, 2011), which suggests that to understand an adaptive trait, we need to consider how it emerges from the complex interplay of activity across different timescales (Rittschof & Hughes, 2018; Trillmich, Günther, Müller, Reinhold, & Sachser, 2015). The HMM builds on such thinking by situating the brain within a broader EST of psychology.

**Fig. 1:**
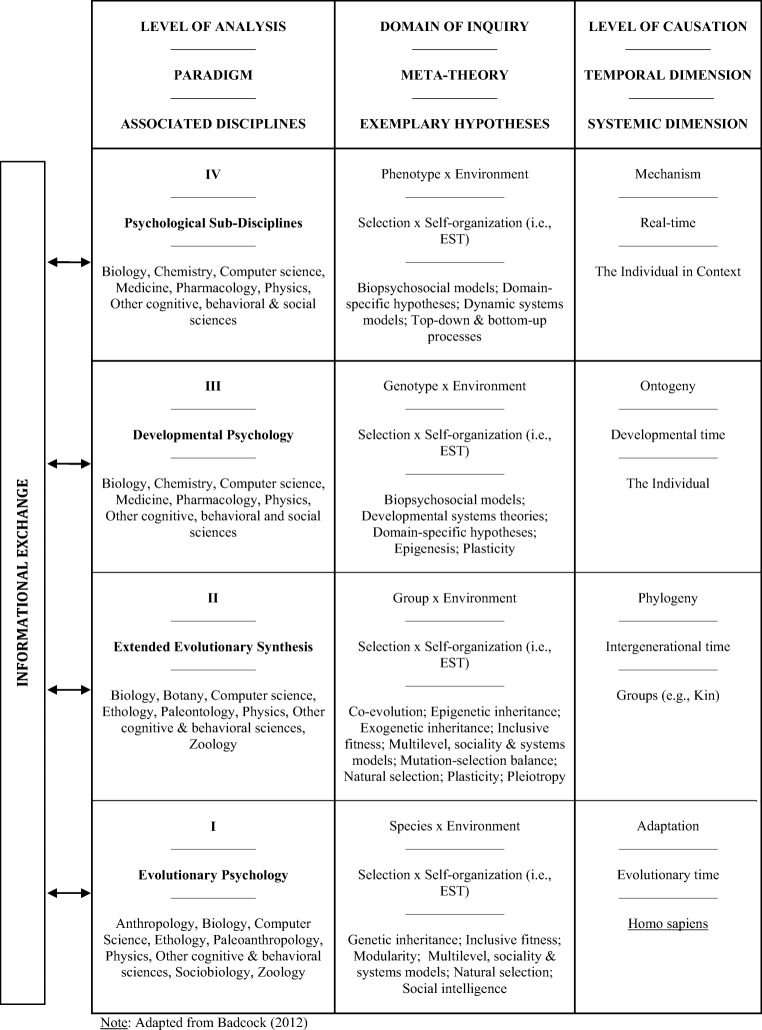
The evolutionary systems theory of psychology. Human phenotypes, cognition and behavior are produced by the complementary influence of selection and self-organization acting across four dynamically coupled levels of causation: adaptation, phylogeny, ontogeny, and mechanism. Psychological paradigms shed light on this process by concentrating differentially on four specific, interrelated levels of analysis: functional hypotheses for adaptive, species-typical characteristics (i.e., evolutionary psychology); explanations for intergenerational, between-group similarities and differences (i.e., *evo-devo* and the extended evolutionary synthesis); explanations for individual development (i.e., developmental psychology); and mechanistic explanations for real-time biobehavioral phenomena (i.e., the subdisciplines). Informational exchange between these paradigms allows researchers to integrate advances across different fields of inquiry and develop new hypotheses, and the nonsubstantive meta-theories of (natural and general) selection and self-organization interpenetrate all four explanatory levels to promote their consilience. For further details, see Badcock (2012)

The EST in question explains the human brain and its relation to our phenotypes, cognition, and behavior in terms of reciprocal interactions between selection and self-organization acting across the four domains of biological phenomena articulated by Tinbergen (1963): adaptation, phylogeny, ontogeny, and mechanism. These domains involve both a temporal dimension (i.e., evolutionary, intergenerational, developmental, and real-time processes, respectively) and a systemic dimension, which relates to the unit over which selection and self-organization operate at each timescale (i.e., all *Homo sapiens*, social groups, the individual over its lifespan, and the individual in context, respectively). As discussed elsewhere (Badcock, 2012), this schematic can be leveraged to organize major paradigms in psychology into four distinct, but complementary levels of analysis (Figure 1).

As shown in the Figure 1, “first-level” analyses are taken up by evolutionary psychology. This is a heterogeneous paradigm that examines the influence of evolutionary processes (particularly natural, kin, and sexual selection) on human psychology and behavior, incorporating influential approaches such as the massive modularity hypothesis, along with sociality, multilevel, and dynamic systems views (Caporael, 2001). To date, the most widely recognized model to emerge from this field is the massively modular mind, which states that the human brain comprises a large collection of species-typical, functionally specialized modules (Buss, 1995, 2016; Tooby & Cosmides, 1992). As we mentioned earlier, these modules are thought to reflect domain-specific adaptations—they have evolved, through the process of natural selection, because they solved distinct adaptive problems by responding to specific input criteria (gleaned from an external environment or other internal processes), and transforming this information into output by influencing physiological activity, activating other mechanisms and/or producing behavior in adaptive ways (Buss, 1995). The validity of this view has attracted a lot of debate; however, because authoritative discussions of this issue are already available (Anderson & Finlay, 2014; Stephen, 2014; Zerilli, 2017), we will not dwell on this debate here. For our part, we certainly endorse the broader evolutionary psychological claim that selection favors the reliable emergence of adaptive, species-typical cognitive and behavioral patterns. We also believe that it is important to distinguish between massive modularity as an explanatory claim about the form and function of the brain and its apparent heuristic value (Klasios, 2014; Stephen, 2014). Regardless of the veracity of massive modularity, evolutionary computational theories continue to guide research in a systematic and highly productive way, providing a wealth of insights into the adaptive significance of our mental processes and behavior (Buss, 2016; Dewsbury, 2009; Dunbar & Barrett, 2007; Frankenhuis & Ploeger, 2007; Machery & Barrett, 2006; Pinker, 1997). Indeed, the convenience of the phenotypic gambit is that it can produce substantive, testable hypotheses of cognition and behavior without requiring a mechanistic explanation for how the brain produces it (Badcock et al., 2016). In this way, the pitfalls of massive modularity by no means vitiate its capacity to motivate meaningful research (Barrett, 2008). As a fully accepted explanation for the adaptive properties of all biological systems, it is also clear that natural selection represents a crucial explanatory principle for psychological inquiry (Badcock, 2012; Burke, 2014; Frankenhuis & Ploeger, 2007; Machery & Barrett, 2006).

As we noted earlier, however, the HMM offers a mechanistic alternative to this hypothesis that enables researchers to retain its heuristic benefits, while dispensing with its problematic, explanatory claims. Specifically, a key difference between massive modularity and our own model is that we do not place any emphasis upon modularity (i.e., functional specialization and computational encapsulation) or domain-specific (vs. domain-general) cognitive processes. By appealing to neomechanistic philosophy (Bechtel, 2008; Craver, 2006), the HMM offers an alternative to modules, in the form of dynamically interacting (and only partially segregated) neurocognitive mechanisms. This approach attributes equal weight to functional segregation and integration; it explains adaptive biobehavioral patterns in terms of dynamic, coordinated interactions between (hierarchically nested and functionally differentiated) neural mechanisms, not separately modifiable, functional modules *per se*. A similar claim has recently been made by Anderson (2014, 2016), who suggests that adaptive behaviors arise from transiently assembled local neural subsystems that are able to respond flexibly to environmental demands, not specialized modules dedicated to solving specific adaptive problems.

Returning to Figure 1, “second level” explanations appeal to the extended evolutionary synthesis (Laland et al., 2015). This is an emerging paradigm that incorporates insights from a number of complementary fields that focus on the dialectical relationship between ontogeny and phylogeny. The best known of these is evolutionary developmental biology (evo-devo), which explores the dynamic ways in which developmental changes within one generation (i.e., ontogeny) relate to changes across generations (i.e., phylogeny) (Hall, 1999, 2003). A key source of heritable ontogenetic variation is *epigenetic inheritance*, where adaptive behaviors and phenotypic modifications are transmitted to subsequent generations without directly altering the genome, supplying new targets for selection (Jablonka & Lamb, 1995, 2005). Another relates to *exogenetic inheritance*, which refers to the set of reliably inherited environmental resources that are necessary for the reproduction of the individual lifecycle, including adaptive, constructed aspects of the ecological niche, and the intergenerational transmission of accumulated cultural information through regimes of imitation, social learning, and explicit teaching (Henrich, 2015; Sterelny, 2012; Stotz, 2017; Tomasello, 2014). Rather than focusing on the endpoints of selection, evo-devo concerns the mechanisms responsible for the origin and development of adaptations over evolutionary time (Oyama, 2000; Ploeger et al., 2008a; West-Eberhard, 2003), thereby synthesizing ultimate, adaptationist explanations with proximate, ontogenetic models to explain evolutionary change (i.e., phylogeny). An increasing number of evolutionary psychologists have embraced the field, resulting in the emergence of *evolutionary developmental psychology* as a distinct subdiscipline (Bjorklund & Pellegrini, 2002; Geary & Bjorklund, 2000). More recently, Heyes (2018, in press) has advocated an approach called *cultural evolutionary psychology*, which concentrates on the ways in which distinctively human (adaptive) cognitive mechanisms emerge from cultural rather than genetic evolution.

“Third-level” explanations relate to developmental psychology. Attempts to unify theorizing in this field have led many to adopt a *developmental systems* approach, driving myriad advances in the study of biological, cognitive, emotional, language, neurological, and personality development (Kelso, 1995; Lewis, 2000; Ploeger et al., 2008b; Thelen & Smith, 1994). This is not surprising, given that dynamical approaches echo the interactionist principles espoused by developmentalists for years (Bronfenbrenner, 1977; Gottlieb, 1991; Karmiloff-Smith, 1992; Lickliter & Honeycutt, 2003; Sameroff, 2010). Self-organization supplies a cohesive, biologically plausible explanation for the appearance of novelty within developing systems, the emergence of order and increasing complexity over time, transition points that permit both structural advances and individual diversification, and our capacity for self-correcting stability and sensitive adaptation to the environment (Lewis & Granic, 1999).

Finally, “fourth-level” mechanistic analyses concern the dynamic ways in which ecobiopsychosocial interactions produce cognition and behavior in real-time. This final tier of analysis is encapsulated by psychology’s subdisciplines, such as cognitive, personality, social, and clinical psychology. By analyzing data collected from particular individuals under specific conditions at a given point in time, subdisciplinary research can be seen as targeting the most proximate, variable level of explanation by exploring the innumerable ways in which unique ontogenetic outcomes (i.e., our phenotypes and behavior) interact with different environments in real-time (Badcock, 2012).

This hierarchical structure of scientific theorizing has long been recognized, particularly in relation to divisions between the physical sciences, biology, psychology, and the social sciences (Henriques, 2011). Analogously, the multilevel structure of different paradigms in psychology can be seen as an expected consequence of scientific inquiry—by asking certain questions, researchers must neglect others; resultant conclusions should be appropriate for the sorts of questions being posed; and we should not undermine a fruitful approach at one level because it fails to address another (Dewsbury, 2009; Marshall, 2013; Scott-Phillips, Dickins, & West, 2011). Such different levels of analysis therefore should be seen as providing distinct, alternate, and valid perspectives on the same whole (Witherington & Lickliter, 2016). At the same time, however, they are complementary and intersect. Researchers can—and often do—exploit this sort of multilevel theoretical organization, because it allows theorizing at one level of analysis to be refined and reinforced through reference to models and findings at others. Clearly, understanding the complexity of the human system also rests on appreciating that different levels of biological activity are reciprocal or co-acting, which means that we need to examine how these levels interact (Marshall, 2013; Scott-Phillips et al., 2011). Indeed, Tinbergen himself emphasized the importance of exploring how his four levels of analysis interrelate (Bateson & Laland, 2013).

To this end, the EST described here adopts a process-oriented approach to Tinbergen’s questions, which is based on the relatively uncontentious claim that all human phenotypes emerge from recursive, dynamically coupled interactions between evolutionary (e.g., natural selection), intergenerational (e.g., epigenetic and cultural inheritance), developmental (e.g., gene-environment interactions), and real-time (e.g., biopsychosocial) processes (Badcock et al., 2019). For instance, in this context, phylogeny refers to the dynamic causal processes responsible for producing heritable changes between generations, not to the outcomes of such processes (i.e., our position on the Tree of Life). At the same time, however, the EST also encapsulates extant research on the outcomes of these processes at all four levels of psychological explanation, begging the question of how the outcomes observed at one level of inquiry emerge from the dynamics at play in others. Such an approach satisfies the remit of evolutionary psychologists by accommodating the influence of natural selection and other evolutionary forces. Conversely, it appeals to the constructivist principles championed by developmentalists, because it recognizes that adaptive phenotypic traits emerge from dynamic interactions between the phenotype and its environment over the course of ontogeny.

So how does this EST of psychology relate to the HMM? Following models in computational neuroethology (Chiel & Beer, 1997; Ramstead et al., 2018a), embodied cognition (Clark, 1999; Wilson, 2002), and enactivism (Gallagher, 2017), the HMM is a theory of the embodied brain that explains neural dynamics, structure, and function in terms of reciprocal interactions between human phenotypes and the environment over four nested temporal scales. In other words, it situates the brain within the multilevel dynamics of the human brain-body-environment system (Bolis & Schilbach, 2018; Gallagher, 2017; Marshall, 2013). This system is produced by a temporal hierarchy of dynamically coupled processes: evolutionary constraints on cognition run through individual development and learning, while effects at these timescales can influence neural evolution in turn (Dickins & Levy, 2001). Next, we briefly explore how this multi-level theoretical framework can be used to explain the hierarchical architecture of the brain.

## Explaining Hierarchically Modular Neural Networks with the HMM

The HMM is a global theory of human neurocognition and biobehavior that follows from the broader meta-theory of EST described above: it explains the hierarchical form and function of the brain in terms of an embodied, complex adaptive system that has been shaped differentially by evolutionary, intergenerational, developmental, and real-time processes, which themselves exhibit circular causality. This perspective aligns with other dynamical proposals, according to which adaptive psychobiological mechanisms (i.e., evolved, epigenetic attractors) emerge from the repeated assembly of reliably recurrent developmental resources produced by reciprocal interactions between an evolutionary history of selection, developmental processes, and situational activities in species-typical, real-time environments (Anderson & Finlay, 2014; Caporael, 2001; Hendriks-Jansen, 1996; Lickliter & Honeycutt, 2003). In humans, an important constraint that extends across all of these timescales is the sociocultural environment, because our survival depends on our ability to leverage cultural information and immersively participate in normative, culturally adapted practices (Gallagher, 2017; Heyes, 2018; Ramstead et al., 2018a, 2018b; Ramstead, Veissière & Kirmayer, 2016). On the basis of these distinctions, the HMM suggests that theories of human brain dynamics should be informed by integrative, multilevel models in psychology that are able to identify both *why* different neurocognitive and biobehavioral patterns are adaptive; along with *how* they emerge from the broader causal processes that act on human phenotypes across various timescales (Badcock et al., 2017; Ramstead et al., 2018a).

This temporal hierarchy of causal mechanisms is arguably manifest in the development and morphology of the brain. Comparative and human studies have shown that the phylogeny of the brain is reflected across nested levels of neural organization—ranging from the genes inherited from our hominid ancestors, to epigenetic transcription factors that shape gene expression, to the synaptic epigenesis of neural networks throughout development, and the long-range connectivity that underpins daily consciousness (Changeux, 2017). Similarly, studies of the maturation of neural networks over childhood and adolescence have shown that human cortical development mirrors phylogeny, progressing from sensorimotor hierarchies akin to those of other mammals through to the recent association areas shared by humans and other primates (Gogtay et al., 2004; Gu et al., 2015). In a review of the comparative literature, Finlay and Uchiyama (2015) describe how the hierarchical organization of the cortex emerges from a rostro-caudal gradient in the duration of neuron production—a phylogenetically variable phenomenon found in every mammal studied to date. They contend that this represents a highly conserved developmental mechanism that directly impacts on brain evolution—producing a progressive increase in both the hierarchical structure and absolute size of the cortex throughout ontogeny and conferring the adaptive advantage of heightened cognitive control among primates and other large brained animals (Finlay & Uchiyama, 2015; also see Badre, 2008). The above findings point directly to the complementary relationship between natural selection and self-organization: selection has canalized early sensorimotor regions that serve as neurodevelopmental anchors, allowing for the progressive self-organization of highly integrated association cortices throughout development that enhance evolvability by responding flexibly to environment change (Anderson & Finlay, 2014; Buckner & Krienen, 2013).

Importantly, we are not the first to apply EST to the brain (Haken, 1996; Kelso, 1995). Of particular relevance, two cardinal properties of complex adaptive systems are that aggregates of interacting units (e.g., modules) are organized in a hierarchically nested manner (Holland, 1995) and that intra-component (e.g., within-module) connections tend to be stronger than inter-component (e.g., between-module) connections, with neighboring components showing stronger connections than distal ones (Eidelson, 1997). It is now widely accepted that this type of hierarchical structure is strongly favored by selection. It enhances evolvability because deleterious changes to a single component of the system are unlikely to affect the system itself, and it allows adaptive novelties to emerge without disrupting global functioning (Sporns & Betzel, 2016). Computer simulations of evolving networks have shown that a hierarchical organization conserves the (spatial, processing, and metabolic) cost of neural connections and adapts faster to new environments than nonhierarchical structures, because it is able to solve problems by recursively combining solutions to subproblems (Mengistu, Huizinga, Mouret & Clune, 2016). Finally, the hierarchical brain is thought to promote “self-organized criticality.” This is a dynamical state poised between completely ordered, stable cycles of activity and highly complex, chaotic ones that optimizes evolvability, because it allows small, extrinsic changes to elicit large, intrinsic reorganizations (Bak & Chen, 1991). Self-organized criticality is a central concept in complexity theory, which has been widely adopted across the sciences to shed light on the dynamics of complex adaptive systems (Bak, 2013). It has also been leveraged to explain the emergence of healthy, optimal, or adaptive human phenotypes and behaviors, whereas deviations from this critical state are thought to lead to aging and disease (Coey, Kallen, Chemero, & Richardson, 2018; Delignières, & Marmelat, 2012). With respect to the brain, the hierarchical segregation of neural networks into distributed neighborhoods has been found to stretch the parameter range for self-organized criticality by allowing subcritical and supercritical dynamics to coexist simultaneously (Hilgetag & Hütt, 2014). Because systems at criticality have optimal information-processing capacities, a structure that extends this critical region is likely to be naturally selected (Hesse & Gross, 2014).

## Explaining the Adaptive Mind: A Variational (Free-Energy) Approach

We have considered a range of perspectives that converge on the idea of a hierarchically structured brain that both instantiates and engenders the complementary relationship between natural selection and self-organization. We also have argued that to understand the brain, one must consider causal interactions between the broader evolutionary, intergenerational, developmental, and real-time influences that shape human phenotypes. However, what is missing from our account so far is a neurobiologically plausible theory that is able to explain *why* the brain is structured in the way that it is and functions in the ways that it does. To address this, we will introduce the free-energy principle (FEP) from computational neuroscience, which can be used to formulate mathematically the dynamics that obtain both within and across all four of Tinbergen’s (1963) levels of causation. Leveraging the resources provided by the FEP allows us to operationalize the HMM and to define its multilevel dynamics formally.

## The Free-Energy Principle

Originally proposed to explain perception, learning, and action (Friston, 2003, 2005), the FEP since has been applied to the evolution, development, form, and function of the brain (Friston, 2010; Friston, Kilner & Harrison, 2006) and, more recently, to the characteristic properties of life itself (Friston, 2013b; Ramstead et al., 2018a). The FEP is a simple postulate with complex ramifications. It states that to remain alive, all living systems must minimize the quantity “variational free-energy” to reduce the entropy (i.e., the decay or dispersion) of their sensory and physiological states. Technically, variational free energy is a formal, information theoretic quantity that limits (by being greater than) the entropy of a generative model entailed by the state of a biological system (e.g., the brain). As noted in our discussion of predictive coding, a generative model refers to a probabilistic mapping from causes in the environment to observed consequences (e.g., sensory data). In this context, entropy refers to the (long-term) average of surprise: the (negative log) probability of sensory samples encountered by an agent (Friston, 2010). Intuitively, organisms expect to remain within their phenotypic states; deleterious deviations from these expectations are in this sense surprising and must be avoided.

The FEP builds on the idea that biological agents are distinguishable from other self-organizing systems because they actively avoid deleterious (surprising) phase-transitions by minimizing the entropy of their sensory and physical states. Living systems are locally ergodic. They revisit a small number of states with a high probability (Friston, 2013b; Schrödinger, 1944). In this context, ergodicity simply refers to the tendency of an organism to revisit continually the same, characteristic phenotypic states. It appeals to the (observable and demonstrable) existence of an attracting set (i.e., pullback attractor) in random dynamical systems, which means that there is a finite probability that the neighborhood of any state will be revisited over a suitably long period of time. That is, the system will appear to be attracted to particular regimes of state or phase space. Notably, this does not imply stationarity or thermodynamic equilibrium. The dissipative processes against which we struggle continue to exist, but our ability to actively reduce surprise allows us to delay their deleterious effects by repeatedly returning to the same, limited set of (unsurprising) phenotypic states. This propensity to minimize surprise (resp. free-energy) is the consequence of natural selection: self-organizing systems capable of avoiding such phase-transitions have been selected over those that could not (Friston et al., 2006). Because the repertoire of functional (i.e., adaptive) states occupied by an organism is limited, mathematically, the probability distribution over these characteristic states has low entropy: there is a high probability that the organism will occupy a small number of states. Thus, an organism’s distal imperative of survival and maintaining functional states within physiological bounds (i.e., homeostasis) translates into a proximal avoidance of surprise (Friston, 2010). Although surprise itself cannot be evaluated, because free-energy imposes an upper limit on surprise, biological systems can indirectly reduce surprise by minimizing their free-energy. To do this, an organism uses sensations and its predictions, which are based on the hierarchical generative model encoded by its internal states (e.g., neuronal activity and connection strengths). Importantly, the FEP generalizes the theory of predictive coding: biological agents actively minimize free-energy by reducing their prediction errors (and indirectly, surprise).

Fundamentally, living beings can minimize surprise either by changing their predictions by altering their internal states (i.e., perception and learning) or by changing their relation with the environment to alter what is predicted (i.e., action). Thus, action and perception operate in a reciprocally causal fashion to maintain homeostasis and optimize an organism’s generative model of the world (Friston, 2010; Friston, Breakspear, & Deco, 2012b). This process is encapsulated by the theory of active inference: the idea that all behavior involves the selective sampling of sensory data to ensure that our predictions are self-fulfilling (Friston, Daunizeau, & Kiebel, 2009; Friston, Daunizeau, Kilner & Kiebel, 2010; Hohwy, 2016).

To summarize, minimizing free-energy simply means inducing an upper bound on surprise by means of predictions, and reducing this bound by optimizing the activity and connectivity in our brains (resulting in action, perception, and learning). Because surprise is mathematically equivalent to the (negative log) of Bayesian model evidence, minimizing free-energy maximizes the evidence for our generative models of the world; it compels us to make Bayesian inferences about our environment. A key extension of this view is that our models of the world are optimized through evolution, neurodevelopment, and learning (Ramstead et al., 2018a). To discuss this further, we turn to the fundamental role of prior beliefs in shaping our predictions, behavior, and the hierarchical structure of the brain.

## Adaptive Priors and the Hierarchical Brain

If each individual is adapted or optimized to their own environment—either at an evolutionary level or on a daily basis due to learning—the expectations (encoded by neuronal form and activity) of each individual must differ. However, we also must inherit some aspect of these expectations to conserve the physical form that entails each generation’s model of its eco-niche (e.g., the way the brain is wired). This brings us to the crucial role of (Bayesian) prior beliefs about the sorts of sensory experiences we expect to encounter in the world (e.g., the fact that we have eyes suggests an environment bathed in light) (Friston, Thornton, & A. Clark, 2012c; Stamps & Frankenhuis, 2016). According to the FEP, species-typical patterns of cognition and behavior can be explained in terms of *adaptive priors*: inherited expectations about the causal structure of the world that have been shaped by selection to guide action-perception cycles towards unsurprising states (e.g., “I will keep moving until I am rewarded”).

Where do these adaptive priors come from? Following neural Darwinism, the FEP explains neurophysiological changes in terms of the influence of selection acting on human phenotypes over evolutionary time. The brain only labels a sensory state as valuable (i.e., unsurprising) if it leads to another valuable state, and selection ensures that an organism moves through a succession of probable states with adaptive value. Thus, natural selection reduces surprise by specifying the value of sensory states through genetic and epigenetic mechanisms, prescribing a small number of attractive states with innate value (i.e., adaptive priors) that minimize surprise by ensuring that an organism seeks out states consistent with its phenotype and environment (Friston 2010). This explains how one generation can pass on to the next what is valuable (expected), without having to prescribe the details of how to attain these valuable states. In short, natural selection is nature’s way of performing Bayesian model selection to minimize the free-energy of phenotypes (i.e., generative models; also see Campbell, 2016).

Notably, the perspective outlined here connects with state-dependent optimality modelling in biology, which concentrates on the properties of biological systems that natural selection is likely to favor under certain ecological conditions (Frankenhuis et al., 2013). This is a Bayesian approach that seeks to understand how state-dependent systems change over time and acquire new information via interactions between their internal states and the environment, with a view to identify the optimal (fitness-maximizing) policy for each possible state of the system (Houston & McNamara, 1999). Akin to the FEP, state-dependent optimality modelling assumes that organisms evolve cognitive and behavioral “rules” (e.g., prior beliefs about states of the world) that perform well on average in their natural environments. Notably, such a view is not tantamount to genetic reductionism: organisms are able to sample cues from the current environment to update the rules that govern their behavior, resulting in posterior beliefs that optimize their models of the local ecology (McNamara, & Houston, 2009; McNamara, Green, & Olssen, 2006; Stamps, & Frankenhuis, 2016). To date, support for these ideas has chiefly been gleaned from simulation studies (Leimar, & McNamara, 2015; Leimar, Dall, Hammerstein, & McNamara, 2016; McNamara, Dall, Hammerstein, & Leimar, 2016; Ramírez, & Marshall, 2017), although there is some preliminary evidence—stemming largely from studies of *Daphia*—that an organism’s genes and inherited physiology provide developmental cues (i.e., priors) that facilitate its flexible adaptation to local ecological conditions (Bell & Stein, 2017; Dall, McNamara, & Leimar, 2015; Hales et al., 2017). From our perspective, this form of optimization reflects free-energy minimization across multiple timescales and has close connections with second-order selection (i.e., selection for selectability), which favors phenotypic traits that optimize an organism’s ability to minimize surprise by enabling it to flexibly adapt to its eco-niche (e.g., phenotypic or developmental plasticity; Houston & McNamara, 1999; Stamps, & Frankenhuis, 2016). These ideas also fit comfortably with the tenets of cybernetics that underpin much of the work on self-organization—particularly the good regulator theorem, which states that any system that can regulate its environment must instantiate a (good or sufficient) model of that environment (Conant & Ashby, 1970; Friston & Buzsáki, 2016; Seth, 2014).

To summarize, the basic tenet of the FEP is that all organisms are compelled to model their world. This follows from the fact that minimizing free-energy implicitly maximizes (Bayesian) model evidence. The ensuing perspective on biological systems says something quite profound: all organisms can be regarded as an embodied statistical model of the environmental niches (i.e., eco-niches) that they inhabit. As such, the FEP not only applies to cortical information processing, but to every element of systemic organization, such as the organization, development, and evolution of the brain (Clark, 2013; Friston & Stephan, 2007). The brain does not just contain a model of the world; it is (one aspect of) a statistical model of the world that is realized by the whole organism—a physical transcription of causal regularities in the environment that is optimized by evolution, neurodevelopment, and learning (Friston, 2013a). The upshot of this is that we should expect to see causal structure in the environment reflected in the anatomical structure of the human brain.

Of particular relevance to the HMM is the emergence of hierarchical connections that speak to lawful statistical regularities conserved over evolutionary timescales (e.g., the laws of physics). For instance, the statistical independence between the identity and location of objects in the visual world suggests an anatomical dissociation between models or representations of the “what” and “where” attributes of (hidden) causes of visual input (i.e., knowing what an object is does not tell you where it is). This is precisely what we see in the distinction between the ventral (“what”) and dorsal (“where”) streams in the cortical hierarchy (Ungerleider & Mishkin, 1982), suggesting that independent environmental causes are encoded in functionally segregated neuronal structures (Friston & Buzsaki, 2016).

Similarly, the architecture of the brain transcribes the inherent hierarchical structure of the world. The explanation for this is fairly simple—any coupled dynamical system will necessarily reflect a hierarchical causal structure that emerges from a separation of temporal scales (Haken, 1983; Murray et al., 2014). This is a cornerstone of many theories in the physical sciences, such as synergetics and the centre manifold theorem—for example, the fast thermal fluctuations at a microscopic scale cannot influence the motion of a massive body at the macroscopic scale. This illustrates a key aspect of hierarchical models: the conditional independence among levels. A hierarchical model is not defined by its connections, but by the absence of connections (i.e., conditional independence). Mathematically, this means that a hierarchy rests upon conditional independencies that are unavoidable in a world that involves a separation of temporal scales. Consistent with this, careful connectivity studies have now evidenced the sparse hierarchical connectivity of the brain (Ercsey-Ravasz et al., 2013; Markov et al., 2014; Mesulam, 2012). This structure can be understood in terms of the hierarchy of temporal scales at which representations evolve. The lowest levels of the brain encode fast fluctuations in the environment associated with sensory processing, whereas higher levels encode more complex causal regularities associated with increasingly slower contextual changes (Friston & Buzsáki, 2016; Kiebel, Daunizeau, & Friston, 2008).

It is worth reiterating that the emergence of structural divisions that reflect the causal structure of the world does not preclude their functional integration. As mentioned, a key property of neural subsystems is their near-decomposability; their information-processing features cannot be fully separated from that of other subsystems (or only abstractly so). Although the specialized processing of a given subsystem depends on short-range connections between its subcomponents, it remains functionally connected to other regions in the network via long-range connections, which facilitates bidirectional message-passing between regions (Park & Friston, 2013). Consistent with this, high-resolution, network-based analyses have shown that different neural subsystems perform discrete cognitive functions, while highly distributed “connector” regions allow for their functional integration by coordinating effective connectivity between these subsystems (Bertolero, Yeo, & D’Esposito, 2015; Taylor, Wang & Kaiser, 2017). Determining the extent to which a given neural region is functionally segregated versus integrated is ultimately an empirical affair, with recent advances in structural and functional brain mapping providing a promising means to explore this issue—as exemplified, in particular, by studies of the human connectome (Sporns, 2011; Sporns, Tononi, & Kötter, 2005; Van Essen et al., 2013).

So far, then, we have discussed the role of natural selection in producing adaptive, phenotypic priors that are reliably passed from one generation to the next. Before we conclude this section, it is important to address the potential charge of genetic reductionism by recognizing that such traits emerge from multiscale interactions between (internal) biological dynamics—ranging from genes, cells, and neural activity, through to organs and the body—and the developmental environment in which such dynamics unfold. Crucially, this includes other human beings and our relations to them, as well as a shared eco-niche (Constant, Ramstead, Veissière, Campbell, & Friston, 2018b). Moreover, we do not mean to suggest that all adaptive priors are genetically inherited. Instead, we are inclined toward an expanded view of inheritance borrowed from evo-devo and the extended evolutionary synthesis, which assumes a dynamic, bidirectional relationship between ontogenetic and evolutionary processes (Laland et al., 2015). This view extends beyond the gene as the single unit of inheritance to incorporate other units of information transmission, including RNA, cells, cytoplasm, organelles, and the extracellular environment (Jablonka & Lamb, 2002). It also incorporates exogenetic forms of inheritance, which involve the intergenerational transmission of cultural information, practices, and niches that shape adaptive cognitive and behavioral policies across generations and over the course of ontogeny (Constant, Ramstead et al., 2018b; Griffiths, 2017; Heyes, 2018; Ramstead et al., 2016; Sterelny, 2012).

With this in mind, we appeal to a multiscale formulation of adaptive priors, only a subset of which are specified genetically. Some adaptive priors will indeed reflect the (epi)genetic inheritance of species-typical traits favored by natural selection. But this is only part of the story. Others will also instantiate *empirical priors*, which leverage information obtained through experience to produce adaptive responses to our eco-niche. The former, putatively “innate” priors will show a strong genetic basis, will tend to be species-typical, and might be found in other species (primates in particular). Among others, exemplary candidates range from the gross morphology of the brain (Friston, 2010), hormonal and neuromodulatory systems (Heyland, Hodin, & Reitzel, 2005; Katz & Harris-Warrick, 1999; McGlothlin & Ketterson, 2008), reward, mood, and affective systems (Adams et al., 2016; Gray, 1972, 1994; Nettle & Bateson, 2012), personality traits (Bouchard & Loehlin, 2001; Gosling, 2001; Nettle, 2006), cognitive biases that emerge early in infancy (e.g., attention toward faces and a phobia of snakes; LoBue & Rakison, 2013; Salva, Farroni, Regolin, Vallortigara, & Johnson, 2011), shared intentionality (Tomasello, 2010; Tomasello & Carpenter, 2007), relational reasoning (Penn, Holyoak, & Povinelli, 2008), and sensitive periods of development (e.g., puberty) that fine-tune our adaptation to different environments across the life course (Fawcett & Frankenhuis, 2015; Frankenhuis & Fraley, 2017; Geary & Bjorklund, 2000).

On the other hand, intergenerational, exogenetic resources allow for the inheritance of adaptive, highly specialized neurocognitive mechanisms (i.e., “cognitive gadgets”; Heyes, 2018) that depend more on cultural evolution and social learning, such as language and mind reading (Heyes, 2018; Heyes & Frith, 2014). As we have discussed elsewhere, the individual also inherits adapted cultural practices from its eco-niche, which have been shaped by other social members to motivate adaptive behavior (e.g., shelters and desire paths; see Constant, Ramstead et al., 2018b; Ramstead, Constant, Badcock & Friston, 2019). Determining the extent to which an adaptive prior reflects innate biobehavioral biases sculpted and scripted by natural selection, or a flexible, adaptive response that relies more on cultural evolution and social learning, is ultimately an empirical matter, although we suspect that in most cases, the development of the one (e.g., social learning) is likely to be intimately tied to the other (e.g., an attentional bias toward faces; also see Heyes, 2018, in press). Our basic point is that adaptive priors arise from the reliable transmission of adaptive (surprise-reducing) policies from one generation to the next. They emerge, differentially, from the evolutionary processes of adaptation and phylogeny and drive developmental and real-time activity at the level of the individual to reduce surprise.

To recapitulate, the FEP asserts that the fundamental imperative for all living systems is to minimize (a free-energy bound on) surprise, which depends on predictions. This idea appeals to ubiquitous procedures in Bayesian statistics – namely, Bayesian inference and model selection, via free-energy minimization (perception, action, learning, and evolution). According to this scheme, natural selection can be seen as performing Bayesian model selection by optimizing phenotypes that are an embodied model of the world they inhabit, exploring the model space proffered by genetic and epigenetic variation—variation that is itself subject to selective pressure—to successively optimize phenotypic models of the eco-niche over evolutionary time (Campbell, 2016; de Vladar & Szathmary, 2015; Harper, 2011). These models are further optimized by niche construction and cultural evolution, which allow flexible, adaptive priors to be shaped by one generation and passed on to the next (Constant, Ramstead et al., 2018b; Ramstead et al., 2019).

In a nutshell, the FEP describes the brain as an adaptive, hierarchically organized neurocognitive system (i.e., a generative model) that functions to minimize prediction errors (and therefore surprise) by seeking to match incoming sensory inputs with top-down predictions. These predictions are constrained by prior beliefs, which allow our physiology and behavior to be optimized by evolution, neurodevelopment, and experience. In the following section, we incorporate these ideas into the HMM by formulating, mathematically, the dynamics of the human brain at each descriptive level specified by the HMM, and the broader EST of psychology to which it appeals.

## The HMM Revisited: Incorporating the Free-Energy Formulation

Earlier, we proposed an EST of the brain (i.e., the HMM) premised on the influence of selection on dynamic interactions between evolutionary, intergenerational, developmental, and real-time processes. There are fundamental points of contact between this theory and the FEP. Both conform to EST by emphasizing the complementary relationship between natural selection and self-organization. They also both assume that natural and cultural selection influence the evolutionary and developmental trajectories of biological systems via the inheritance of distinctive neurocognitive patterns (e.g., adaptive priors) that guide cognition and behavior in adaptive ways. Finally, they assert that neural processing mechanisms are hierarchically organized, interact in a recursive fashion, and involve both specialization and integration. To synthesize these models and precisely define the HMM, we will now return to the schematic of the EST of psychology described earlier.

Beyond the fact that they are both hierarchical models that draw from the principles of EST, the FEP converges with the HMM in two fundamental ways. We have already noted in our treatment of the FEP that although each individual is adapted or optimized to his or her own eco-niche—meaning that everyone is different—the inheritance of adaptive priors suggests the existence of species-typical phenotypic traits. Like the HMM—and the EST to which it appeals—this denotes a systemic dimension that extends from all *Homo sapiens* to a specific individual in real-time.

**Fig. 2:**
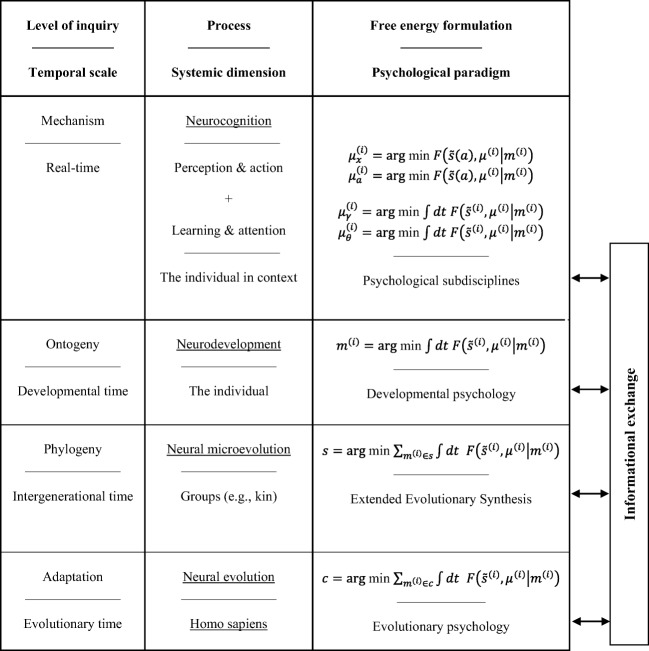
The hierarchically mechanistic mind. In this scheme, *F*$\left(\tilde{s},{\mu}^{(i)}|{m}^{(i)}\right)$ represents the free-energy of the sensory data (and its temporal derivatives) $\tilde{s}$(*a*) and states *μ* of an agent *m*^(i)^ ϵ *s* that belongs to a subgroup *s*ϵ*c* of class *c*. Action (*a*) governs the sampling of sensory data, and the physical states of the phenotype (*μ*) encode beliefs or expectations (and expectations about the mean of a probability distribution). Reproduced from Badcock et al. (2019)

The second, related similarity is that both models appeal to recursive causal interactions between different temporal scales. As displayed in Figure 2, this process can be expressed formally according to the timescales over which free-energy minimization optimizes the state (perception), configuration (action), connectivity (learning and attention), anatomy (neurodevelopment), and phenotype (evolution) of biological agents that belong to a given class (species).

As shown in Figure 2, the FEP can be used to formulate dynamics for phenomena at each of the levels of analysis entailed by the HMM. Specifically, Level IV (i.e., mechanistic) explanations relate to neurocognition, which entails two sets of interacting processes. The first of these includes perception and action, which optimize neuronal and neuromuscular activity to suppress an individual’s prediction errors (resp. free-energy) based on generative models of fluctuating sensory data (Friston, 2011). The second entails learning and attention, which involve the optimization of synaptic gain and efficiency over seconds to hours to encode the precision of prediction errors and causal structure in the sensorium (Friston, 2011). Level III (i.e., ontogenetic) explanations relate to neurodevelopment, which involves generative model optimization through activity-dependent pruning and the maintenance of neuronal connections that are transmitted epigenetically (Friston, 2011). Level II (i.e., phylogenetic) explanations refer to the optimization of the average free-energy over generations of individuals belonging to a particular subgroup (e.g., kin) of a given class (i.e., conspecifics), via the exo- and epi-genetic transmission of generative models. Finally, adaptation can be described as the optimization of the average free-energy over time and individuals of a given class (i.e., conspecifics) via the influence of selective pressure on their generative models or adaptive priors.

In summary, the HMM offers an integrative theory of the embodied human brain for the psychological sciences, based on an EST of psychology that synthesizes evolutionary and developmental explanations for the brain, mind, and behavior (Badcock, 2012). The HMM also leverages the resources of the FEP to formally operationalize evolutionary, developmental, and real-time influences on neural structure and function (Badcock, 2019). The resulting perspective depicts the brain as an evolved, self-organizing system comprising hierarchical networks of neural subsystems that function collectively to minimize the entropy or decay of our sensory and physiological states. More precisely, the HMM defines the human brain as a (situated and embodied) complex adaptive system that actively minimizes the variational free-energy (and therefore entropy) of (far from equilibrium) phenotypic states via self-fulfilling action-perception cycles, which are mediated by dynamic interactions between hierarchically organized, functionally differentiated neurocognitive mechanisms (Badcock et al., 2019). This structure instantiates adaptive priors, which have been shaped by evolutionary forces to guide our action-perception cycles toward adaptive (i.e., unsurprising) states. In closing, we turn now to the implications of this model for theorizing and research in psychology.

## Using the HMM as a Research Heuristic

Thus far, we have proposed an interdisciplinary model of the embodied brain that bridges major schools of thought in neuroscience and psychology; furnishes both an ultimate, evolutionary explanation for human phenotypes and a proximate, process theory of our mental processes and behavior (i.e., adaptive free-energy minimization); and explains cognition in terms of hierarchical neural dynamics that minimize prediction error (resp. surprise) via bidirectional message passing between differentially integrated subsystems. This model is best understood as a first-level hypothesis predicated on the meta-theory of EST. It therefore resembles other widely known schemes of the brain, such as predictive coding and massive modularity, in that it can be used as a systematic heuristic to generate unique, integrative hypotheses from which more specific, testable predictions can be derived.

At this juncture, it is important to address potential concerns about our attempt to provide a unifying theory of the brain. As Anderson (2014) points out, the sheer diversity of cognitive and behavioral capacities observed in *Homo sapiens*, and the surfeit of theories that we have developed to explain them, suggests that a single theory of neural dynamics is unlikely to be helpful and runs the risk of obscuring important differences between highly distinctive neurocognitive processes. In response to this concern, we echo Clark (2013) by appealing to the historical debate between the “neats,” who maintained that intelligence is underpinned by a small number of general principles, and the “scruffies,” who thought that intelligence arises from a motley collection of improvised solutions to new ecological problems. Clark (2013) suggests that the theory of predictive coding is capable of accommodating both of these camps: it provides a generalizable account of global brain function that extends across all neural processes; conversely, it fails to specify the precise and manifold ways in which the brain implements this scheme. Similarly, the FEP satisfies the “neats” by providing a single imperative that is realized by all of the quantities that can change in a living system. However, it also allows us to think of cognition as “scruffy,” because it only imposes relatively modest (information-theoretic) constraints on neural structure and function, leaving ample room for evolution and development to produce a wide array of idiosyncratic (free-energy minimizing) strategies. In much the same way, the HMM subsumes the FEP as a generalizable principle of human neural and biobehavioral dynamics, but it also demands recourse to substantive research in psychology (and other allied sciences) to elucidate the distinctive ways in which this principle manifests in humans (Badcock et al., 2019; Ramstead et al., 2018a).

We also believe that the explanatory value of any unifying theory ultimately depends on its capacity to generate substantive, testable hypotheses that are able to explain a diversity of concrete phenomena in detail. To this end, both predictive coding and the FEP have already proven to be enormously fruitful (Clark, 2013; Hohwy, 2013; Friston, FitzGerald et al., 2017a). We hope that the HMM follows suit. Indeed, although it is important to remain skeptical of unifying theories, we do not believe that this should preclude attempts to synthesize paradigms in a meaningful way that promotes consilience and offers new pathways to scientific progress. We join many others by arguing that different theories can act synergistically by creating new ways to improve our understanding of the mind and behavior (Barrett, 2008; Caporael, 2001; Frankenhuis et al., 2013; Kenrick, 2001; Ploeger, Van Der Maas, & Raijmakers 2008a, 2008b).

Another likely concern is whether our model can be tested directly. In response to this issue, we note that the HMM rests on several empirically tractable claims that render the theory itself open to scientific scrutiny. By way of illustration, consider the ways that hierarchical predictive coding has been tested empirically, which provide an informative exemplar of how our own theory might be put to the test. Here, we focus on an elegant study by Harrison and colleagues that examined the role of backward (i.e., top-down) connections in the visual cortex in suppressing prediction error (Harrison, Stephan, Rees, & Friston, 2007). This experiment involved measurements of evoked responses to predictable and unpredictable visual stimuli to test the hypothesis—derived from the theory of hierarchical predictive coding—that evoked responses in early (lower) visual areas would be reduced for predictable, relative to unpredictable, stimuli. Because showing reduced responses to predictable stimuli does not allow for the inference that this reduction is mediated by backward connections, sparse stimuli were used that excited retinotopically mapped responses beyond the range of horizontal connections in the primary visual cortex (V1)—given that any component motion of a single stimulus that could be predicted by other stimuli can only be “seen” by higher visual areas with larger receptive fields (i.e., in V2 or higher), differences in V1 responses (due to predictability) must be mediated by backward connections from V2 or higher. Accordingly, incoherent and globally coherent sparse stimuli were presented to participants every second or so, while hemodynamic responses were measured using fMRI. As predicted, V1 responses to predictable (relative to unpredictable) stimuli were significantly reduced, evidencing error suppression by backward connections. This study offers a clear example of how exploiting known anatomical characteristics of brain connectivity (in this case, the range of the receptive fields of V1 versus higher visual areas) can inform neuroimaging studies (for example) to demonstrate the suppression of errors at lower cortical areas by predictions from higher regions (see also Friston, 2018). Of course, this is only one way to test for predictive processing via hierarchical message passing; others have been reviewed extensively elsewhere (Adams, Huys & Roiser, 2016; Adams, Stephan, Brown, Frith, & Friston, 2013; Bastos, 2012; A. Clark, 2013, 2016; Hohwy, 2013). Nevertheless, we think it suffices to show how one of the central assumptions of the FEP—and by extension, the HMM itself—can be tested empirically.

The question also arises as to what kind of observation would be able to *falsify* the HMM. Clearly, robust evidence against the nested, hierarchical organization of different neural regions would call into question our central architectural claim. One way to gather such evidence would be to use structural and functional connectivity studies to compare the anatomical segregation and/or integration of lower-order sensorimotor regions with that of higher association areas (Sporns, 2013; Rubinov, & Sporns, 2010). More broadly, as a model that commits to the influence of *selective pressure* on neural structure and function, one would expect to observe species-typical, homologous characteristics in the basic wiring and gross morphology of the brain (at least at some scale). Evidence, for example, of substantial cultural variation in human neuroanatomy across all scales of interest would not sit well with this assumption. Cases such as these show that some of the foundational claims of the HMM can, in principle, be examined empirically, and potentially falsified as well. Robust evidence against any one of these claims would cast into doubt the HMM itself.

That being said, we believe the more important question is not whether the HMM itself might be falsified, but instead, how it might be used to generate testable, second-order hypotheses about specific phenomena, which can be compared with hypotheses derived from competing theories (e.g., massive modularity). We echo our earlier point that the HMM is as much a framework to guide theorizing and research as it is a model of the human brain (Badcock et al., 2019). In this spirit, it is worth accentuating the heuristic benefits of the broader EST to which the HMM belongs. Following seminal frameworks like Tinbergen’s (1963) four research questions and Marr’s (1982) tri-level approach to information processing, the HMM clearly specifies the different explanatory levels that should be targeted by researchers in psychology. More precisely, it emphasizes the need to exploit complementary consistencies between theories and findings that have emerged from evolutionary psychology, the extended evolutionary synthesis, developmental psychology, and the subdisciplines (Badcock, 2012; Ramstead et al., 2018a). This heuristic is intended to promote sophisticated, multilevel hypotheses that integrate insights drawn from disparate fields of inquiry and inspire new avenues for research. The upshot of this modeling strategy is that it should maximize the evidence for such hypotheses by requiring researchers to consolidate supportive findings spanning all four of Tinbergen’s levels of inquiry (Ramstead et al., 2018b). Encouragingly, dynamical methods also are available that allow us to analyze the ways in which different levels of activity interact, such as the use of dynamic optimization to explore how selection influences human development over time (Frankenhuis et al., 2013) and computer simulations to examine how adaptive policies influence the dynamics of social behavior (Kenrick, Li, & Butner, 2003).

Although we lack the scope to explore this issue properly, it should be noted that the HMM has important implications for neuroscientists (Badcock, Friston, & Ramstead, 2019). As mentioned, different organisms instantiate distinct “embodied models” of their species-typical eco-niches (Friston, 2011). This means that the FEP can accommodate all forms of biotic morphology and behavior, but such a generalizable principle only affords limited insight into the specific features of a given species (Clark, 2013; Ramstead et al., 2018b). To explain the human brain, we still require content—a substantive, evolutionary account that sheds light on the particular adaptive solutions responsible for the embodied models of *Homo sapiens*, while also capturing the proximate processes that influence every phenotype (Clark, 2013; Ramstead et al., 2018a). Thus, substantiating the FEP demands recourse to psychology, because it explicitly identifies the complex, multilevel processes responsible for human cognition and behavior in particular (Badcock et al., 2019).

The HMM addresses this issue by situating the FEP within a broader EST of human phenotypes. In this respect, it answers repeated calls for a dialectical relationship between neuroscience and psychology, where insights gleaned from one are actively exploited to inform and constrain theorizing and research in the other (Barrett, 2009; Crone & Ridderinkhof, 2011; Pfeifer & N.B. Allen, 2012, 2016; Piccinini & Craver, 2011). More particularly, our model encourages methodological approaches that are able to identify the psychological factors responsible for different patterns of neural activity in different contexts, such as analyses of large databases of task-based fMRI activation studies to characterize the functional fingerprints of specific neural regions across different task demands (Anderson, 2014), or the development of *cognitive ontologies* that systematically map relationships between specific cognitive functions and hierarchical neural dynamics (Poldrack, 2010; Price & Friston, 2005). Furthermore, approaches in developmental psychology can be leveraged to explore the dynamic ways in which human ontogeny differentiates error-minimizing policies between individuals, such as theoretically informed longitudinal designs that combine neuroimaging work on brain maturation with appropriate biological, psychological, and social measures to examine how different developmental contexts produce stable biases in perceptual inference and behavior (Crone & Ridderinkhof, 2011; Huys & Dayan, 2009). Capitalizing on the wealth of comparative, cross-cultural, computational, and dynamical approaches in evolutionary psychology also promises to shed light on the (epi- and exo-)genetic mechanisms that underlie our species-typical adaptive priors (Badcock et al., 2016). Finally, dynamical methods, such as computer simulations and computational models, allow us to examine directly how different levels of activity interact (Chiel & Beer, 1997; Frankenhuis et al., 2013; Friston, Stephan, Montague, & Dolan, 2014; Ramstead, et al., 2017), enabling neuroscientists to explore how the phenomena highlighted by psychologists reflect adaptive free-energy minimization under multifarious evolutionary, intergenerational, developmental, and real-time conditions. The outcomes of such analyses can then be confirmed through reference to real-world observations (Ramstead et al., 2018a, 2018b). Having briefly discussed the potential implications of our theory for neuroscience, we will now take a closer look at how it might be leveraged by researchers in psychology.

## Implications for Theorizing and Research in Psychology

A particularly important corollary of the HMM is the need to extend the principle of free-energy minimization to all domains of psychological inquiry. Traditionally, applications in neuroscience have concentrated on fourth-level, mechanistic phenomena, such as perception (Kiebel, von Kriegstein, Daunizeau, & Friston, 2009), action (Friston et al., 2010), attention (Feldman & Friston, 2010), and learning (Friston, 2008). Direct support for the FEP has mainly been gleaned from computer simulations (Friston et al., 2009; Friston et al., 2010; Friston, FitzGerald et al., 2017a), studies of the visual system (Keller, Bonhoeffer & Hübener, 2012; Kok, Jehee, & de Lange, 2012; Markov et al., 2014), and analyses of microcircuits in the brain (Bastos et al., 2012; Shipp, 2016). Typically, researchers in this area have used computer simulations, fMRI, and/or EEG to apply (computational) dynamic causal models of interactions between hierarchically organized cortical regions to explain neural responses to unpredictable stimuli (Friston et al., 2006), along with increasingly sophisticated phenomena, such as insight and curiosity (Friston, Lin et al., 2017b; Moulin & Souchay, 2015). Despite such progress in neuroscience, psychologists have been relatively slow to exploit the explanatory power of the FEP.

This is not to say that it has gone unrecognized. Indeed, since the relevance of the FEP to all fields of psychological inquiry was first recognized (Badcock, 2012), others have taken up this theory to cast new light on human mental life (Clark, 2013, 2016; Hohwy, 2013)—tackling subjective phenomena such as anxiety (Hirsh, Mar, & Peterson, 2012), emotion (Barrett & Simmons, 2015; Joffily & Coricelli, 2013; Seth, 2013), illusions (Brown et al., 2013), delusions and hallucinations in schizophrenia (Fletcher & Frith, 2009), and consciousness itself (Hobson, & Friston, 2014; Wiese, 2018). Notably, the FEP also lends itself to methods that are already familiar to psychologists, such as the P300. This is an event-related potential that can be used as a noninvasive, temporally sensitive proxy of surprise, allowing researchers to capture dynamic error suppression over time by measuring trial-by-trial fluctuations in P300 amplitudes (Kolossa, Fingscheidt, Wessel & Kopp, 2013; Mars et al., 2008).

Indeed, one of the main virtues of the HMM is that by invoking the FEP, it provides a new way of thinking about cognition and behavior that can be fruitfully extended across all levels of psychological inquiry. Promising parallels between the FEP and major traditions in psychology certainly suggest as much (Friston, 2010). Of particular note, both the FEP and HMM resonate with key principles of ecological psychology. Based on the pioneering works of Gibson (1966, 1979), Barker (1968), and Bronfenbrenner (1977, 1979), this is a relational approach that focuses on the ways in which cognition and behavior emerge from reciprocal organism-environment relations over time (see Heft, 2001, 2013). Central to this paradigm is the notion of an affordance, which broadly refers to a relation between the abilities and expectations of an organism and aspects of its material world (Chemero, 2009; Gibson, 1979). Consistent with the FEP, which describes the processes responsible for policy selection and adaptive behavior, ecological psychologists advocate a pragmatic, action-oriented approach to cognition (Heft, 2013). The notion of organism-environment reciprocity also clearly connects with the idea that human phenotypes instantiate a generative model of their eco-niche (Constant, Ramstead et al., 2018b; Friston, 2013b). Elsewhere, we have explored in some depth how the FEP appeals to other major foci in this field, such as hierarchical, multiscale interactions (Ramstead et al., 2018a; Ramstead et al., 2019), as well as the environmental affordances that guide our behavior, the influence of sociocultural dynamics on individual cognition and enculturation, and the behavioral settings, physical artifacts, and normative practices laid down by social groups (Constant, Ramstead et al., 2018b; Ramstead et al., 2016).

It is worth noting that the FEP is also commensurate with representationalism. This is because free-energy is defined in relation to an approximate posterior probability distribution—a Bayesian belief about hidden causes in the environment. This is important for two reasons. First, such beliefs are quintessentially representational, because they are “about” the causes of sensory input. Second, it means that the level of analysis afforded by the FEP can be cast in terms of (posterior and prior) beliefs in a straightforward way that map quite naturally to established constructs in psychology (Badcock et al., 2017; Carhart-Harris & Friston, 2010).

Active inference has also been applied to reinforcement learning in cognitive and behavioral psychology. Approaches in this area typically operate under the framework of expected utility theory. They are based on the idea that the selected action maximizes the expected utility (or reward) associated with the outcomes expected following that action. The expected utility of a policy is determined by combining the agent’s subjective probability assessments of states of the world with its utility rankings over outcomes. These utility rankings are modelled as value or cost functions that represent the agent’s preferences (Ramsey, 1931; Von Neumann & Morgenstern, 1945). A major problem with this approach is that value functions are constructed in such a way that appealing to them as an explanatory account of the origin of the values or preferences of agents, and of their corresponding optimization schemes, is circular (Friston & Ao, 2011; Friston, Adams, & Montague, 2012a; Friston et al., 2015; Pezzulo, Cartoni, Rigoli, Pio-Lopez, & Friston, 2016). Specifically, classical schemes define optimal behavior as the policy that maximizes the probability of obtaining valuable outcomes, but value functions, in a circular fashion, are defined as objective functions that describe optimal behaviors (Friston, Shiner et al., 2012d). Although value functions can be used to represent such preferences and describe decisions based on them, what is lacking is an account of how these preferences originate and change over time.

The FEP addresses this issue by formulating the utility or value of a policy in terms of the (adaptive and empirical) priors that organisms acquire over several nested timescales. This solves some of the deep problems that attend classical approaches by absorbing classical value functions into prior preferences over outcomes, which are based on the biological imperative to minimize surprise. Central to this approach is the idea that living systems are not simply in the game of reducing free-energy in the present moment; they must also choose actions that reduce *expected free-energy*; i.e., the expected surprise or uncertainty associated with the outcomes of action (Friston et al., 2017; Friston, Rosch, Parr, Price, & Bowman, 2018). Expected free-energy can be decomposed into *epistemic value* and *pragmatic value*, which connects with the exploration-exploitation trade-off in ethology, game theory, and economics (Cohen, McClure & Yu 2007; Ishii, Yoshida & Yoshimoto 2002). Epistemic value corresponds to the expected information gain that results from an action, leading to explorative behaviors that seek out observations that resolve uncertainty (e.g., foraging to find prey). Pragmatic value refers to prior preferences over future outcomes (i.e., those that are likely to minimize surprise) and drives goal-directed, exploitative behavior. This latter construct is basically equivalent to expected utility in classical theories, where utility or reward is expressed in terms of a log probability. In other words, an outcome with high utility is simply an outcome that the agent, *a priori,* expects to encounter.

In this context, the exploration-exploitation dilemma is resolved by the relative contributions of epistemic and pragmatic value to expected free energy, and consequently, to policy selection. When an agent is uncertain about the state of affairs in the world, it will engage in epistemic or exploratory behavior to gain information and thereby enable pragmatic action in the future. When the agent is confident about environmental states, pragmatic value will dominate and the behavioral policy switches to an exploitative one that seeks to fulfill goals directly (Friston et al., 2015; Friston et al., 2018). In short, active inference avoids the circularity of traditional approaches by replacing rewards with prior beliefs about how the world should unfold. These are, in effect, normative beliefs motivated by the evolutionary and ethological imperative to minimize surprise. Survival does not depend on seeking out rewards per se; it depends on avoiding surprising states, which, *a priori*, have low utility (Friston, Adams et al., 2012). This scheme has been used to solve benchmark problems in optimal control theory, such as the mountain car problem (Friston et al., 2009), and has since gleaned support from simulation experiments and studies of choice behavior in humans (Friston et al., 2013; Schwartenbeck, FitzGerald, Dolan, & Friston, 2013; Schwartenbeck et al., 2015).

To refer back to the four levels of psychological analysis described earlier, the FEP has also inspired a number of models that appeal directly to the subdisciplines. Here, we will concentrate on social psychology. Clearly, the sheer complexity of interpersonal exchanges—and the evolutionary imperative to navigate them successfully—alludes to the adaptive benefits of a brain that has been designed by selection to minimize (social) uncertainty. In this vein, it has been proposed that predictive coding is able to explain mentalizing—our ability to estimate the intentions, knowledge, and beliefs of others (Frith & Frith, 2012; Kilner, Friston, & Frith, 2007). Arguably, we use these estimations to predict others’ behaviors and then update our estimations based on the resultant prediction errors (Frith & Frith, 2012; Veissière, 2018). On the other hand, predictive processing has also been applied to atypicalities in mentalizing, particularly by work on autism (Constant, Bervoets, Hens, & Van de Cruys, 2018a; Palmer, Lawson, & Hohwy, 2017).

Elsewhere, the FEP has been leveraged to explain self- and other-representations. Under this model, the function of inferred representations—of both the self and others—is to minimize interpersonal surprise by enabling us to predict and optimize the likelihood of preferred (i.e., unsurprising) social outcomes (Moutoussis, Fearon, El-Deredy, Dolan, & Friston, 2014a). For instance, evaluating one’s past successes in a particular social context can be used to estimate future outcomes in similar situations, while beliefs about others’ traits (e.g., “cheater”) allow us to predict their intentional mental states and interpersonal responses (e.g., “cheating”). Self-representations also guide behavior by serving as their own desirable outcomes (e.g., “I would like to be respectable”). Thus, both self- and other-representations can be understood as heuristics (i.e., prior beliefs) that reduce uncertainty and facilitate optimal behavior in social interactions. Consistent with the HMM, these prior beliefs vary across individuals as a function of development and (epi)genetics, but also incorporate implicit social, cultural and evolutionary norms and goals (Moutoussis, Fearon et al., 2014a). Following the FEP, prior beliefs about likely social outcomes are weighted by their precision (i.e., one’s confidence in those beliefs) and are successively updated with experience (Moutoussis, Trujillo-Barreto, Deredy, Dolan, & Friston, 2014b). By way of demonstration, Moutoussis, Trujillo-Barreto and colleagues (2014b) have used simulations of a multi-round Investor-Trustee game to show how beliefs about one’s own prosocial preferences and the traits of an opponent are updated during iterated play and produce changes in interpersonal behavior (i.e., entrusting different portions of one’s wage to an unknown investor).

Intriguingly, the FEP has also been extended beyond social cognition to explain interpersonal behaviors, such as dyadic conversation. Following active inference, it has been proposed that communication enables two actors to resolve the uncertainty involved in simultaneously inferring each other’s mental states by adopting a shared narrative (i.e., a generative model), which is intermittently generated by both actors (Friston & Frith, 2015b). This narrative allows each actor to predict the sensations caused by the other (i.e., by listening) and to predict sensations caused by the self by articulating the narrative (i.e., by speaking) (Friston & Frith, 2015b). In other words, two actors successfully predict both themselves and each other by attenuating and augmenting their incoming sensory signals (i.e., by speaking and listening, respectively), thereby minimizing their mutual prediction errors (Friston & Frith, 2015a). Interestingly, the turn taking mandated by this sort of mutual prediction requires inference about agency (i.e., determining whose turn it is), which speaks to a close relationship between dyadic coupling and a sense of agency and selfhood. By producing a reciprocal exchange of sensory signals, the shared narrative induces a generalized synchrony between the neuronal states that generate predictions in both actors (i.e., neural coupling), allowing them to change each other’s minds and facilitate learning (Friston & Frith, 2015b). This process has been demonstrated via simulations of birdsong (Friston & Frith, 2015a), while a viable means to examine it in humans would be to use brain activation studies to look for intersubject correlations in patterns of brain activity between speakers and listeners (Schoot, Hagoort & Segaert, 2016).

Finally, the FEP has recently been applied to large-scale, sociocultural phenomena. In their work on cultural affordances, Ramstead and colleagues describe how shared expectations among members of a social group become encoded neuronally as high-level priors through individuals’ immersive participation in social practices over the course of ontogeny (Ramstead et al., 2016; Veissière, 2018). These norms and conventions help us make sense of the world and guide cooperative action in situationally appropriate ways, reducing uncertainty (resp. free-energy) at both the individual and group level by regulating joint attention and shared intentionality (Ramstead et al., 2016). In a similar vein, Clark (2013, 2016) has speculated that sociocultural systems minimize prediction error for the members of social groups through a process of cumulative, communally distributed reasoning. Under this scheme, material artifacts, institutions, and cultural practices can be seen as products of sociocultural generative models that facilitate adaptive (i.e., valuable) responses to shared environments.

Although the relevance of the FEP to social psychology has only started to become clear, the applications described above highlight its ability to contribute meaningfully to the field. More particularly, the elegant idea that we operate together to minimize collective uncertainly stands to cast new light on classical phenomena, such as conformity, compliance, and the self-fulfilling prophecy. It also lends itself to a range of methodologies, such as multibrain imaging studies that are able to unpack the hierarchical neural dynamics that minimize shared prediction error, along with computer simulations and social network analyses to test computational models of how group behavior reduces mutual surprise (Badcock et al., 2017; Ramstead et al., 2018a). Conversely, evidence-informed theories drawn from evolutionary, developmental, and social psychology can facilitate progress in the active inference literature by illuminating the distinct patterns of social cognition and behavior that we should expect to observe in humans. As such, the heuristic benefits of synthesizing the FEP with social psychology are likely to run both ways.

On the other end of the meta-theoretical hierarchy, its emphasis on adaptive priors suggests that the FEP can readily accommodate evolutionary psychology. Take, for example, the cheater-detection module, which is thought to have evolved to facilitate the detection and avoidance of social contract violations (Cosmides & Tooby, 1992). Conceivably, this phenomenon might reflect an adaptive prior that minimizes specific prediction errors (i.e., absence of reward) by instantiating expectations of an increased probability of cheating in contexts involving uncertain social contracts for mutual gain (e.g., exchange relationships; Fiske, 1991). By combining standard ways to test this hypothesis (e.g., modified versions of the Wason selection task; Cosmides & Tooby, 1994) with neuroimaging or electrophysiological measurements that gauge error suppression in contexts involving responses to predictable versus unpredictable stimuli (e.g., trial-by-trail fluctuations in P300 amplitudes; Kolossa et al., 2013), evolutionary psychologists stand to provide support for their models by shedding light on the hierarchical neural dynamics responsible for cognitive adaptations. Critically, the HMM also suggests that adaptive psychobiological patterns should be attributed to adaptive priors instead of separately modifiable, functionally specialized modules. This idea still allows for some form of adaptationism, but it avoids the pitfalls of massive modularity by adopting a neurobiologically plausible view that explains cognition and behavior in terms of dynamic, hierarchical patterns of neural activity.

Ultimately, we believe the advantage of the FEP over other predictive coding approaches is that much like psychology, it encompasses behavior as well as cognition, the body as well as the brain, along with human evolution and development. Its roots in EST further suggests that it shares fundamental similarities with psychological paradigms that emphasize the complementary relationship between natural selection (resp. adaptation) and self-organization (resp. phylogeny and ontogeny). Finally, it takes these dynamical principles and applies them across multiple levels of causation, arguably recapitulating the meta-theoretical structure of psychological science (Badcock, 2012). Indeed, although those unfamiliar with the FEP may find its technical details inaccessible, under simplifying (statistical) assumptions, it can be reduced to a simple rubric that is readily applicable to all fields of psychological inquiry: cognition and behavior work together to resolve uncertainty and minimize surprise (i.e., active inference). Expressed otherwise, everything we think and do stems from the biological imperative to optimize our predictions about the way the world unfolds and to behave in ways that confirm them (Hohwy, 2016). As we have argued elsewhere, we believe this simple idea can provide a single common language to synthesize and explain diverse findings in the field (Badcock, 2012; Badcock et al., 2019; Friston, 2013b; Ramstead et al., 2018a).

Nevertheless, an outstanding question is whether the rubric of free-energy minimization offers more to psychologists than “just so stories” (Allen, 2018; Hohwy, 2015; Van de Cruys et al., 2014). Whether it generates enough useful insights that cannot already be supplied by existing paradigms remains to be seen. There also are clear translational obstacles to be expected when applying a formal theory of the brain to the sorts of subjective, behavioural, and social phenomena of interest to psychologists. In a species known for its biases toward novelty and misattribution, for its sensation seeking and openness to experience, we also suspect that the FEP may strike some as counterintuitive. If organisms act to minimize surprise, how is it that they can seek out novel and unexpected stimuli—a form of behavior that is clearly very central to human life (think of jazz and horror movies)?

Notably, this issue has been addressed in two complementary ways. The first concerns what has been called the “dark room problem” (Friston, Thornton et al., 2012; Sims, 2017; for a principled solution to this, see Parr & Friston, 2017). The problem is simple: if organisms act to minimize surprise, why don’t they seek out a dark, stimulus-impoverished room and stay there? The answer to this question appeals to the adaptive priors that are characteristic of humans, which specify allostatic and homeostatic set points; i.e., stable, bounded ranges in the values of blood pressure, heartrate, blood sugar levels, etc. To remain alive, organisms must keep these variables within phenotypic bounds, which entails adaptive action. As such, organisms do not seek out globally unsurprising states (like a dark room where nothing happens), but instead seek outcomes that are unsurprising, relative to their adaptive priors. Evolution, development, and learning generate prior beliefs about the sorts of states an agent should expect to occupy, including preferences over outcomes and the specific actions that it might perform in the future to remain within its phenotypic bounds. Because these priors are embodied in physiological and morphological states, which entail behavior-inducing set points, organisms will mostly be on the move, acting in the world to satisfy their set points and remain within phenotypic bounds.

The second response to this issue appeals to the notion of epistemic value, which we introduced earlier. To elaborate, the idea of surprise minimization does not preclude active exploration or an appreciation of novelty but suggests that such behaviors are a valuable means by which to minimize expected free-energy, i.e., to select adaptive actions that minimize expected surprise or uncertainty (Schwartenbeck et al., 2013). This appeals to the fact that, under the FEP, the information gained by sampling the world (e.g., through a visual saccade) is quantified as the uncertainty resolved by that observation. In this framework, the most informative observation is simply the one that resolves the greatest uncertainty; as they plan their actions and forage for information, organisms will tend to seek out the most salient sensations in the service of improving their models of the world. In other words, the FEP suggests that human agents (and more generally, any agent that minimizes expected free energy through policy selection) will seek out novel stimuli that afford the opportunity to resolve uncertainty through action (Friston, FitzGerald, Rigoli, Schwartenbeck, & Pezzulo, 2016; Pezzulo et al., 2016). This intrinsic imperative to resolve uncertainty about the world is driven by the epistemic value of a particular action policy (Parr & Friston, 2017; Ramstead et al., 2019). This is usually framed in terms of salience in treatments of visual search (Itti & Baldi, 2009). Exactly the same mechanics apply to the parameters of our generative models—rendering novelty-seeking a natural consequence of minimizing expected free-energy (Parr & Friston, 2017). This is sometimes treated in terms of artificial curiosity, intrinsic motivation, and knowledge-seeking (Barto, Mirolli, & Baldassarre, 2013; Friston, Lin et al., 2017b; Oudeyer & Kaplan, 2007; Schmidhuber, 2006, 2010). Returning to more proximate states, it is worth noting that the FEP has also been leveraged to explain pleasant surprises (Friston & Friston, 2013; Vuust et al., 2018). In this context, pleasure is thought to be experienced because we move from a state of high to relatively low free-energy. Consider, for example, the punchline of a joke, which elicits the most pleasure the moment it is understood and the right kind of narrative reveals itself (depending, mind you, on the joke; see Joffily & Coricelli, 2013; Westbury, Shaoul, Moroschan, & Ramscar, 2016).

Altogether, we believe that the FEP provides a compelling explanation for biobehavioral dynamics that has now attracted enough theoretical and empirical support across the cognitive sciences to promote its widespread adoption in psychology. To this end, we would strongly encourage the use of active inference as an overarching principle to synthesize diverse findings in the discipline. Following Holland (1998), a promising way to examine such integrative theories empirically is to use dynamical computer simulations to explore how the FEP explains situated biobehavioral patterns under the various sorts of (evolutionary, developmental, and real-time) conditions highlighted by psychologists (Frankenhuis et al., 2013; Kenrick et al., 2002; 2003). The outcomes of such analyses might be confirmed through experimental research, potentiating a fruitful dialectic between computational analyses and real-world observations.

Arguably, the multilevel heuristic that we advocate here also affords considerable protection against overly conjectural hypotheses. As mentioned earlier, the strength of an evolutionary systems approach in psychology is that it demands hypotheses rallied around theoretical advances and empirical support gleaned from all four of Tinbergen’s levels of inquiry. Unlike many of the traditional paradigms in psychology, such as *nativism* and *constructivism*, it requires theories that exploit consistencies across various levels of psychological research to encapsulate the full continuum of ultimate and proximate human processes, along with complex interactions between them (Badcock, 2012; Badcock et al., 2019). This implies that simply applying the FEP to explain a given phenomenon—and describing how it manifests in the brain—is not enough. The HMM also requires us to support such models with research that extends across all four domains of psychological inquiry. By imposing such stringent criteria, it minimizes the risk of highly speculative “just so” stories (Ramstead et al., 2018b).

We would also add that even without an understanding of neural dynamics, the HMM is likely to have considerable utility for researchers across the sub-disciplines. In this respect, psychologists can proceed by asking three complementary research questions: (1) What, if any, is the adaptive function of a given trait? (2) What are the evolutionary, intergenerational, developmental, and real-time processes that produce it? and (3) How does it manifest in beliefs, expectations, or predictions that drive mutually reinforcing cycles of action and perception in order to fulfill them? This is not to say, however, that any hypothesis derived from the HMM is complete without a concomitant model of how the phenomenon of interest emerges from distinct patterns of hierarchical neural dynamics. It is, after all, a theory of the brain. With these considerations in mind, we will now exemplify the full modeling strategy promoted by the HMM by considering its only application in the literature to date: namely, to the human capacity for depression.

## Applying the HMM to depression: an exemplar

Although there is a wealth of Darwinian models of depression, a central premise of many of these is that normative levels of depressed mood reflect an adaptive strategy that conserves (and typically reallocates) an individual’s energy and resources in unpropitious social environments (Allen & Badcock, 2006; Durisko, Mulsant, & Andrews, 2015). Such “resource conservation” models suggest that depression is caused by aversive social outcomes (e.g., exclusion, defeat, or loss) that were typically associated with a loss of control over interpersonal contexts that played a critical role in ancestral fitness (Gilbert, 2006). A model that subsumes many of these views is the *social risk hypothesis*. This suggests that depression reflects an evolved, biobehavioral strategy that prevents the further deterioration of interpersonal relationships by: (1) increasing individuals’ cognitive sensitivity to environmental cues of social risk; (2) reducing their behavioral propensity for taking social risks; and (3) generating signaling behaviors that attract social support and defuse aggressive or competitive encounters (Allen & Badcock, 2003, 2006).

The idea that depression reflects an evolved response to adverse social conditions resonates with extensive evidence across Tinbergen’s remaining levels of analysis. The intergenerational transmission of susceptibility to depressive disorders due to deleterious social environments is widely documented (Vialou, Feng, Robison, & Nestler, 2013; Weissman et al., 2005), with animal and human studies showing that exposure to social stressors (e.g., low maternal care) can produce heritable epigenetic changes that confer risk for disorder by heightening stress reactivity (Meaney, 2001; Sun, Kennedy, & Nestler, 2013). Developmentally, early exposure to social stress (e.g., parental neglect) is thought to heighten depressive vulnerability by leading to hyperactivity of the HPA axis and up-regulating proinflammatory immune responses (Gold, 2015; Slavich & Irwin, 2014). Furthermore, behavioral and neuroimaging studies suggest that the risk of depressive onset rises markedly in adolescence because of an increased sensitivity to social threats in this period (Lambin, Murawski, Whittle, & Fornito, 2017; Silk, Davis, McMakin, Dahl, & Forbes, 2012). Finally, research across the subdisciplines has furnished convincing evidence that the precipitants and correlates of depression directly relate to adverse social contexts (Gotlib & Hammen, 2014; Joiner & Coyne, 1999). Consistent with the social risk hypothesis, depressed mood is associated with improved social problem-solving (Forgas, 2017) and an increase in the accuracy of social inferences (e.g., depressive realism; Moore & Fresco, 2012), along with a specific attentional bias towards socially threatening stimuli (Allen et al., 2001; Mathews, Ridgeway, & Williamson, 1996). Moreover, behavioral correlates of depression, such as social withdrawal and reassurance-seeking, reflect explicit attempts to elicit support and defuse potential conflict (Hagen, 2011; Sloman & Gilbert, 2000). Other studies have provided direct support for the social risk hypothesis itself (Badcock & Allen, 2003, 2007; Dunn, Whelton, & Sharpe, 2012).

Having briefly outlined a multilevel EST of depression, the next step is to consider how this proposed adaptive response relates to free-energy minimization. Although it is important to acknowledge that depression is a heterogeneous phenomenon that stems from multiple etiologies, we have recently suggested that depressive reactions commonly reflect a risk-averse adaptive prior that minimizes uncertainty in the social world when sensory cues indicate a high degree of socioenvironmental uncertainty and an increased probability of aversive interpersonal outcomes, such as rejection or defeat (Badcock et al., 2017). Following the social risk hypothesis, we have proposed that depression instantiates a biobehavioral “better safe than sorry” strategy that causes adaptive changes in perception (e.g., anhedonia and a heightened sensitivity to social risks) and action (e.g., avoidant or cautious social behaviors such as withdrawal). Arguably, epigenetic and ontogenetic mechanisms support this function by sensitizing the individual to volatility in the social world when developmental insults indicate a high probability of aversive interpersonal outcomes, producing hyperreactive stress response systems that increase risk for disorder by heightening sensitivity to social prediction errors and negative interpersonal events (Badcock et al., 2017). In line with active inference, this can generate ongoing depressive behaviors that seek to confirm negative biases, creating a self-fulfilling prophecy (i.e., high predictability) that springs from mutually reinforcing patterns of cognition and behavior (Chekroud, 2015).

At this point, it is necessary to distinguish between depression as an affective state (e.g., sadness), as an adaptive mood state, and as a chronic, pathological state. In the active inference literature, moods are viewed as hyperpriors that constrain short-term emotional fluctuations by encoding higher-level predictions about their long-term average, which suggests a separation of temporal scales when responding to prediction errors (Clark, Watson, & Friston, 2018). In the case of depression, this means that uncertain or negative social outcomes will, on average, be predicted with high precision, suppressing responses to proximate, positively valenced stimuli (Badcock et al., 2017). In other words, depression is associated with high levels of expected (socioenvironmental) free-energy. If the depressive response performs its adaptive function properly, the consequent changes in the social environment should facilitate the revision of expected free-energy (i.e., the depressive hyperprior) over time, thereby alleviating depressed mood. For example, depressed individuals display help-seeking behavior, which is likely to prompt others to engage in care taking (with the effect of reducing socioenvironmental volatility). However, when the depressive response fails to resolve or worsens social stress, this will perpetuate the depressive state by confirming the hyperprior, and the individual is at risk of entering a self-fulfilling dysregulated state, which falls beyond the normal range of adaptive functioning (also see Chekroud, 2015).

Incidentally, this idea concords with recent empirical work arising from a symptom network approach to psychopathology. According to this view, depression is characterized as a complex dynamic system of causally interacting (psychological, behavioral, and biological) symptoms, which can generate self-perpetuating feedback loops that reinforce the disordered state over weeks, months, and even years (Borsboom, 2017; Borsboom & Cramer, 2013). Support for this view has been gleaned from simulation studies (e.g., Cramer et al., 2016), along with network analyses of symptom dynamics in depressed individuals over time (Beard et al., 2016; Epskamp et al., 2018; van Borkulo et al., 2015). One avenue that has yet to be explored in this area concerns the ways in which symptom patterns vary according to individual differences in biological, psychological, and sociocultural factors (Borsboom, 2017). Our analysis suggests that social stressors may be particularly important foci for future research.

**Fig. 3:**
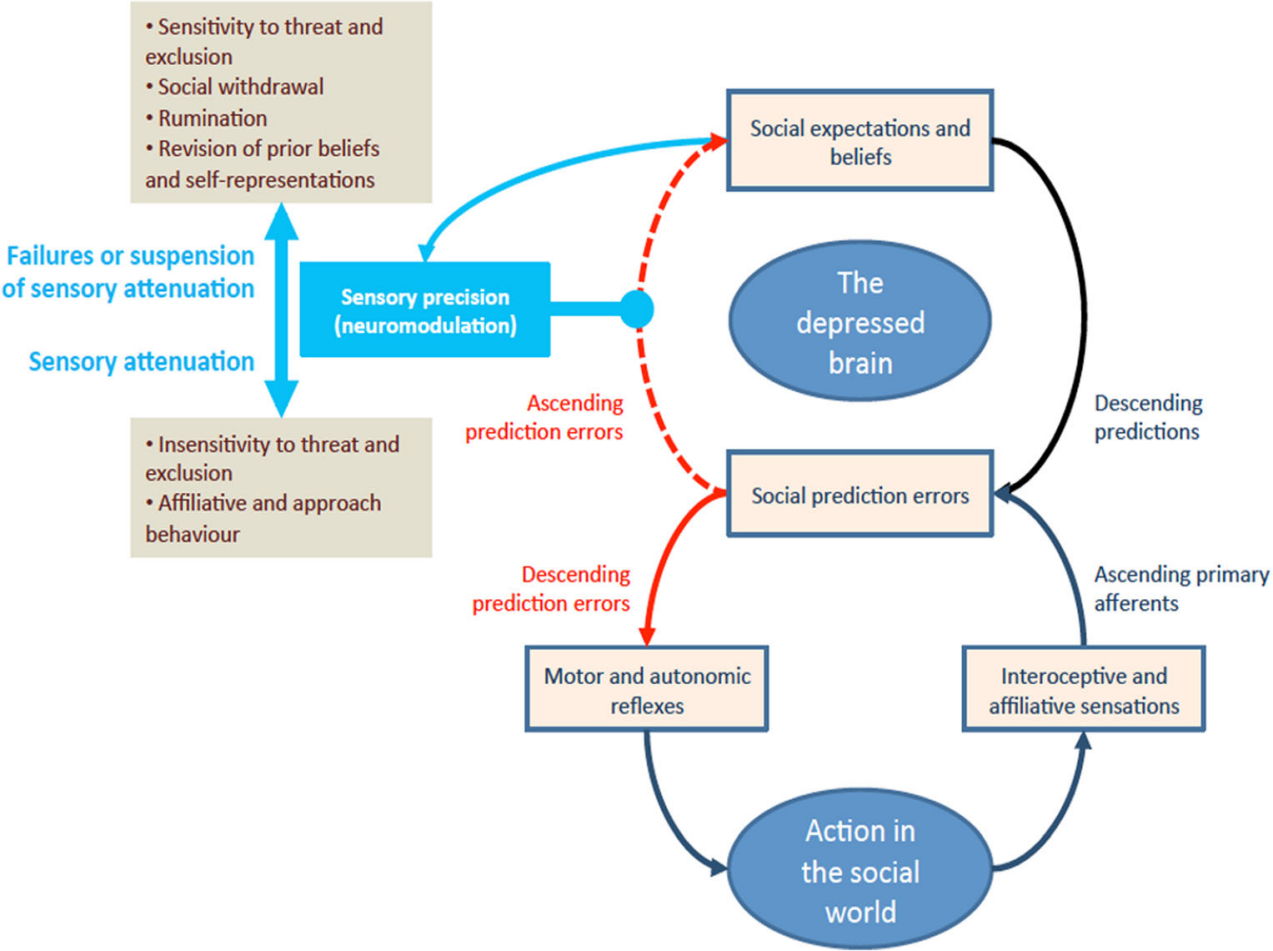
Schematic of the depressed brain. In active inference, action is mediated by motor and autonomic reflexes that are driven by descending (proprioceptive and interoceptive) prediction errors, such that reflexes resolve sensory prediction errors. Action is accompanied by the attenuation of (the precision of) ascending prediction errors. However, if prediction errors cannot be resolved through action this sensory attenuation is suspended—enabling ascending prediction errors to revise posterior beliefs and provide more appropriate top-down predictions. Under this model, adaptive states of depression entail an increase in the precision of (bottom-up) social (interoceptive and affiliative) prediction errors, which enables perceptual inference and learning about the causes of (aversive) social stimuli. This increase in precision heightens sensitivity (i.e., attention) to socio-environmental cues, while reducing confidence in (top-down) social predictions. Cognitively, this is reflected in the suspension of goal directed behavior (e.g., anhedonia), increased rumination about self-other relations, and an attentional bias toward aversive social cues. In pathological depression, we suppose a persistent failure of sensory attenuation that induces aberrant prior beliefs about the probability of social rewards, producing negative expectations (e.g., pessimism, low self-worth). This failure can be pernicious and self-maintaining, because it resolves uncertainty by soliciting sensory evidence that social rewards are unlikely and precluding exploratory behaviors with uncertain outcomes. In other words, both adaptive and aberrant depressed states reduce uncertainty in the social world by suppressing confident or acquisitive (reward-approach) behaviors, and by generating signalling behaviors that seek reliable support (e.g., reassurance seeking) and defuse conflict (e.g., submissive behaviors). Reproduced from Badcock et al. (2017)

The fourth and final step of our modeling approach concerns how this adaptive prior is implemented neurobiologically. Following Price and Drevets (2012), we have argued that depression is associated with dysfunction of the “extended visceromotor system,” which mediates emotional processing through the regulatory effects of the medial prefrontal cortex on visceromotor output, via connections with the amygdala, ventral striatum, hypothalamus, and other subcortical regions. Importantly, many of the regions across this network regulate motivation and reward-approach behaviors and are responsible for processing social threats and rewards (Kupferberg, Bicks, & Hasler, 2016; Nestler et al., 2002; Rushworth, Mars, & Sallet, 2013). We have proposed that this system responds to socioenvironmental volatility by increasing sensitivity to (i.e., the precision of) social prediction errors, causing changes in top-down expectations that produce social withdrawal and increase attention to social stimuli, thereby motivating further avoidance of interpersonal stressors (Badcock et al., 2017; Figure 3). These neurocognitive patterns are adaptive when the consequent changes in mood state and behavior reduce uncertainty in the social world and lead to reengagement with this environment when socioenvironmental volatility abates (which should partly result from depressive behaviors; Allen & Badcock, 2003). This depressive response becomes maladaptive, however, when there are primary structural or functional deficits in the (limbic) visceromotor brain network—produced, for example, by chronic social stress—leading to erroneous interoceptive prediction error signals that promote ongoing hypersensitivity to interpersonal cues, often despite any improvements in the social domain (Barrett & Simmons, 2015). Alternatively, the development of the PFC throughout adolescence can increase vulnerability to depression by allowing for the formation of abstract interpersonal goals that—when frustrated by rejection or failure—can engender depression by suppressing the brain’s reward system and undermining our confidence in the precision of our beliefs about our social behavior (Davey, Yücel, & Allen, 2008). Thus, depressed states can either result from changes in limbic neural threat systems or from the dysregulation of executive prefrontal systems (Badcock et al., 2017; Pfeifer & Allen, 2012).

In closing, we would note that our model of depression is capable of motivating new subdisciplinary research programs. One way to test this hypothesis in the laboratory would be to employ psychophysiological indices of error suppression (e.g., trial-by-trial fluctuations in P300 amplitudes) to compare the neural responses of depressed versus nondepressed samples presented with unpredictable social stimuli (Badcock et al., 2017). Otherwise, social psychologists could explore whether mildly depressed individuals preserve instrumental social relationships more effectively than nondepressed individuals by using paradigms from experimental social psychology, along with longitudinal studies of interactions between changes in mood, social behavior, social networks, and sociometric status (Allen & Badcock, 2003). In personality psychology, our model calls for studies on how traits like neuroticism—an endophenotype that confers vulnerability to affective disorders (Badcock et al., 2011)—underlie individual differences in the precision weighting of social prediction errors and increase risk for psychopathology by heightening reactivity to social stress. More generally, it also requires researchers to couple predictive coding approaches with observational and longitudinal methods in psychology to explore how genetic, epigenetic, and environmental influences shape the development of individual differences in neurophysiological responses to volatility in the social world. As discussed elsewhere, our EST of depression also has important ramifications for diagnosis and treatment in clinical psychology (Badcock et al., 2017). Although these examples are illustrative rather than exhaustive, it should be clear that applying the rubric of the HMM to complex phenomena, such as depression, not only promotes integrative, evidence-based hypotheses, these can then be leveraged to drive new research programs across the subdisciplines.

## Conclusions

Our purpose in this article was to present a unifying, transdisciplinary theory for understanding human psychology and behavior. The HMM is a first-order hypothesis about the structure, function, and dynamics of the human brain. It explains the hierarchical architecture of neural networks, it offers both a formal and substantive explanation of neurocognition and biobehavior that demands the synthesis of psychology and neuroscience, and it harmonizes theorizing and research across the manifold domains of psychological science. Of course, it remains to be seen whether our theory inspires new and productive research questions or facilitates collaboration between psychologists and neuroscientists. The challenge of developing sophisticated theories of human brain dynamics that synthesize the FEP with all four levels of psychological analysis is obviously burdened by complexity, and it is one that will require ongoing collaboration between cognitive and behavioral scientists from diverse fields of inquiry. Such pursuits are certainly worth the effort—although they are perched high on the tree of knowledge, the fruits of such labors are undoubtedly the sweetest.
